# Exploring native *Scutellaria* species provides insight into differential accumulation of flavones with medicinal properties

**DOI:** 10.1038/s41598-022-17586-1

**Published:** 2022-08-01

**Authors:** Blake Costine, Mengzi Zhang, Shweta Chhajed, Brian Pearson, Sixue Chen, Satya Swathi Nadakuduti

**Affiliations:** 1 Department of Environmental Horticulture, University of Florida, Gainesville, FL USA; 2 Department of Environmental Horticulture, Mid-Florida Research and Education Center, University of Florida, Apopka, FL USA; 3 Department of Biology, Genetics Institute, University of Florida, Gainesville, FL USA; 4 Plant Molecular and Cellular Biology Program, University of Florida, Gainesville, FL USA

**Keywords:** Metabolomics, Biochemistry, Plant sciences

## Abstract

*Scutellaria baicalensis* is a well-studied medicinal plant belonging to the Lamiaceae family, prized for the unique 4′-deoxyflavones produced in its roots. In this study, three native species to the Americas, *S. lateriflora*, *S. arenicola,* and *S. integrifolia* were identified by DNA barcoding, and phylogenetic relationships were established with other economically important Lamiaceae members. Furthermore, flavone profiles of native species were explored. 4′-deoxyflavones including baicalein, baicalin, wogonin, wogonoside, chrysin and 4′-hydroxyflavones, scutellarein, scutellarin, and apigenin, were quantified from leaves, stems, and roots. Qualitative, and quantitative differences were identified in their flavone profiles along with characteristic tissue-specific accumulation. 4′-deoxyflavones accumulated in relatively high concentrations in root tissues compared to aerial tissues in all species except *S. lateriflora*. Baicalin, the most abundant 4′-deoxyflavone detected, was localized in the roots of *S. baicalensis* and leaves of *S. lateriflora*, indicating differential accumulation patterns between the species. *S. arenicola* and *S. integrifolia* are phylogenetically closely related with similar flavone profiles and distribution patterns. Additionally, the *S. arenicola* leaf flavone profile was dominated by two major unknown peaks, identified using LC–MS/MS to most likely be luteolin-7-O-glucuronide and 5,7,2′-trihydroxy-6-methoxyflavone 7-O-glucuronide. Collectively, results presented in this study suggest an evolutionary divergence of flavonoid metabolic pathway in the *Scutellaria* genus of Lamiaceae.

## Introduction

*Scutellaria* is a genus found within the Lamiaceae, or mint family, which consists of popular herbal plants including mints, basil, rosemary, and lavender. *Scutellaria* genus, commonly known as skullcap, includes approximately 360 species distributed worldwide from Europe, the U.S., and East Asia^1^. *Scutellaria baicalensis* (Baikal skullcap) has historically been used in Traditional Chinese Medicine (TCM) and is by far the most studied *Scutellaria* species^2,3^. Huang-Qin, a herbal preparation from the root tissue of *S. baicalensis*, is used to treat diarrhea, dysentery, hypertension, hemorrhaging, insomnia, inflammation, and respiratory infections^3^. Furthermore, several in vitro studies using the root extracts of *S. baicalensis* illustrated anti-proliferative and apoptotic activity against colon cancer cells, brain tumor cells, acute lymphocytic leukemia, lymphoma, and myeloma cell lines^2,4–12^.

*Scutellaria* genus is rich in flavones, which are flavonoid metabolites derived from the phenylpropanoid biosynthetic pathway. All flavonoids typically have the same basic skeleton consisting of two 6-C rings (A and B rings) linked by a 3-C bridge that usually forms a third ring (C ring) as in flavones. The medicinal properties of *S. baicalensis* have been attributed to unique 4′-deoxyflavones that lack a 4′-hydroxyl group on their B-rings, produced primarily in the roots of this species^2,4–6,13–15^. The specialized flavone biosynthetic pathway for these unique bioactive 4′-deoxyflavones found in roots of *S. baicalensis*, including baicalein, wogonin, and their glycosides baicalin and wogonoside, respectively*,* has been deciphered^3^. These bioactive flavones are reported to promote apoptosis in tumor cells in vitro with low toxicity in healthy cells and inhibit tumor in vivo in varied mouse tumor models^5,15,16^. In addition to *S. baicalensis, S. barbata* has also been well studied, the dried herbs of which are documented to have medicinal properties in TCM to treat a spectrum of ailments including various cancers especially inhibiting the growth of breast cancer cells^17,18^.

The whole genome has been sequenced for both *S. baicalensis* (2*n* = 18)^19^, and *S. barbata* (2*n* = 26)^20^, and comparative genome analysis of both species revealed a whole-genome duplication event in *S. barbata,* resulting in quantitative chromosomal variation between the species. Furthermore, a functional divergence of genes between the two species resulted in chromosome expansion and species-specific evolution of flavone biosynthetic pathway^20^. American skullcap, *S. lateriflora,* has also been used by native Americans to treat various nervous disorders and as a sedative for insomnia^21,22^. Interestingly, different parts of the plant have been reported to be used for each of these species, roots for *S. baicalensis* and dried aerial tissues for *S. barbata* and *S. lateriflora,* due to differential accumulation of bioactive metabolites supporting the evolution of the flavone biosynthetic pathway. Recent exploration of a few native species identified distinctive organ-specific accumulation of flavones compared to *S. baicalensis* and *S. barbata*^23^. Several *Scutellaria* species native to the Americas have yet to be explored for their flavone metabolites. We hypothesized that exploring native *Scutellaria* species at the DNA, phylogenetic and metabolite levels will identify natives of potential medicinal importance and elaborate the diversity profiles of *Scutellaria* species.

In this current study, three native species of *Scutellaria,* including *S. arenicola*, *S. integrifolia*, and *S. lateriflora,* distributed across the state of Florida, U.S., were investigated. The objective was to elucidate morphological, phytochemical, and genetic differences as compared to the well-studied *S. baicalensis*. DNA barcoding was used for species identification and to evaluate the species' genetic diversity. Subsequent phylogenetic analysis was carried out to elucidate the evolutionary relationships amongst the *Scutellaria* species and other Lamiaceae members of economic importance. Flow cytometry was used to calculate the total nuclear DNA content of each species. High-performance liquid chromatography (HPLC) was used to identify and quantify eight known flavones found in *S. baicalensis* and compare their localization patterns amongst leaf, stem, and root tissues of the native *Scutellaria* species. Significant unknown peaks detected during the HPLC flavone analysis were identified with subsequent HPLC fractionation and LC–MS/MS.

## Results

### Morphological characterization of the *Scutellaria* species used in this study

**Figure 1:**
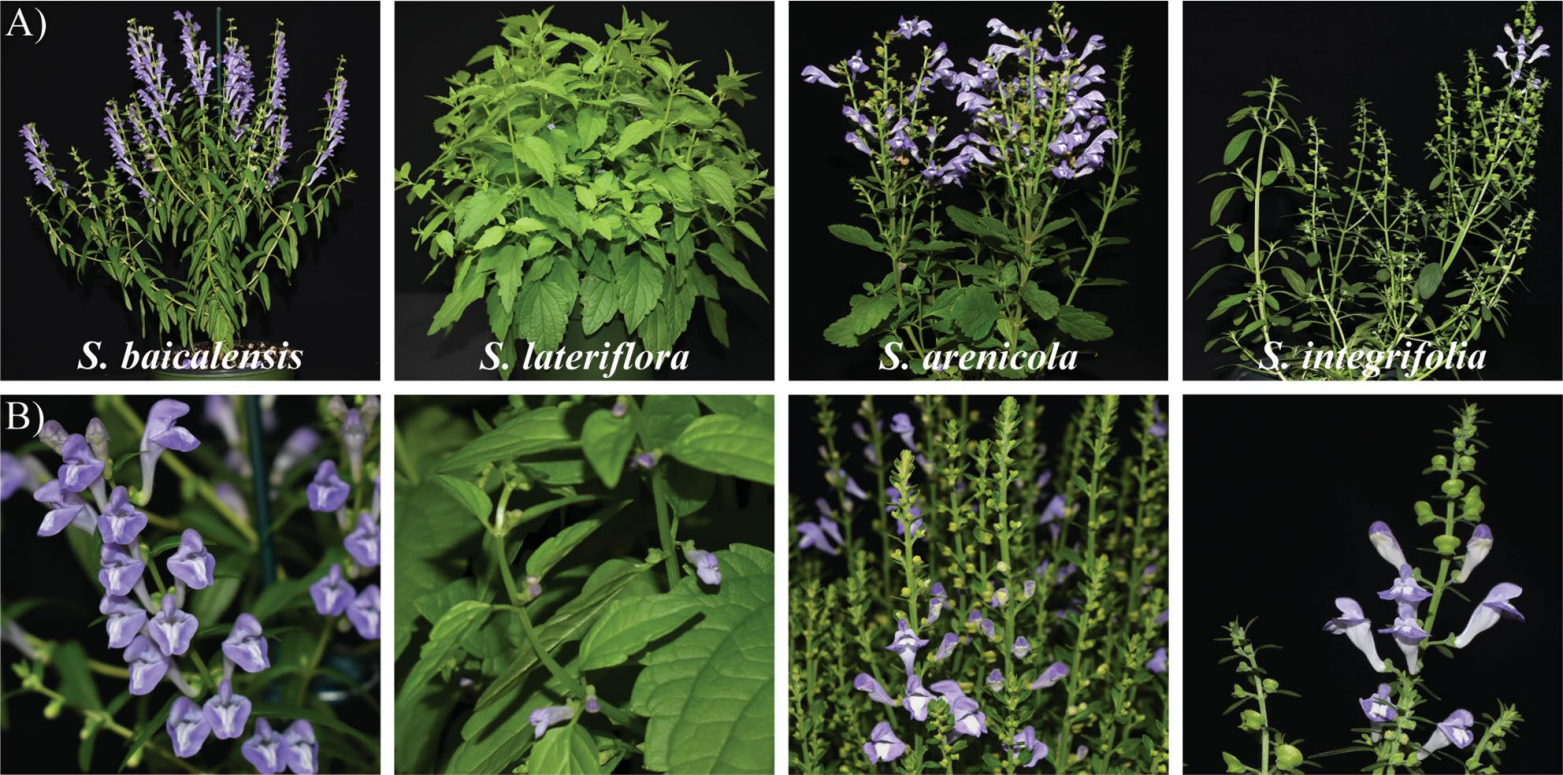
Morphology of the *Scutellaria* species used in this study. Characteristic features of the Lamiaceae family including angular stems, simple opposite leaves, and lobed flower petals. (**A**) plant growth habit and (**B**) flower morphology and size variations observed from the species in (**A**).

The morphology of *S. baicalensis*, *S. lateriflora*, *S. arenicola,* and *S. integrifolia* varied widely. All *Scutellaria* species retained the characteristic features of Lamiaceae family members, including four-angled stems, simple opposite leaves, and five-lobed and two-lipped calyces. However, leaf shape and leaf margins, flower size, and overall canopy structure easily differentiated the four species (Fig. 1A). Flowers of the *Scutellaria* species in this study had blue flowers with three fused petals forming the upper lip and two fused petals forming the lower lip consistent with Lamiaceae family. However, the size of the flowers was markedly different between species, especially the flowers produced by *S. lateriflora* that were visibly smaller, and the other three species were more comparable (Fig. 1B).

### Identification of *Scutellaria* species by DNA barcoding and phylogenetic analyses

**Figure 2:**
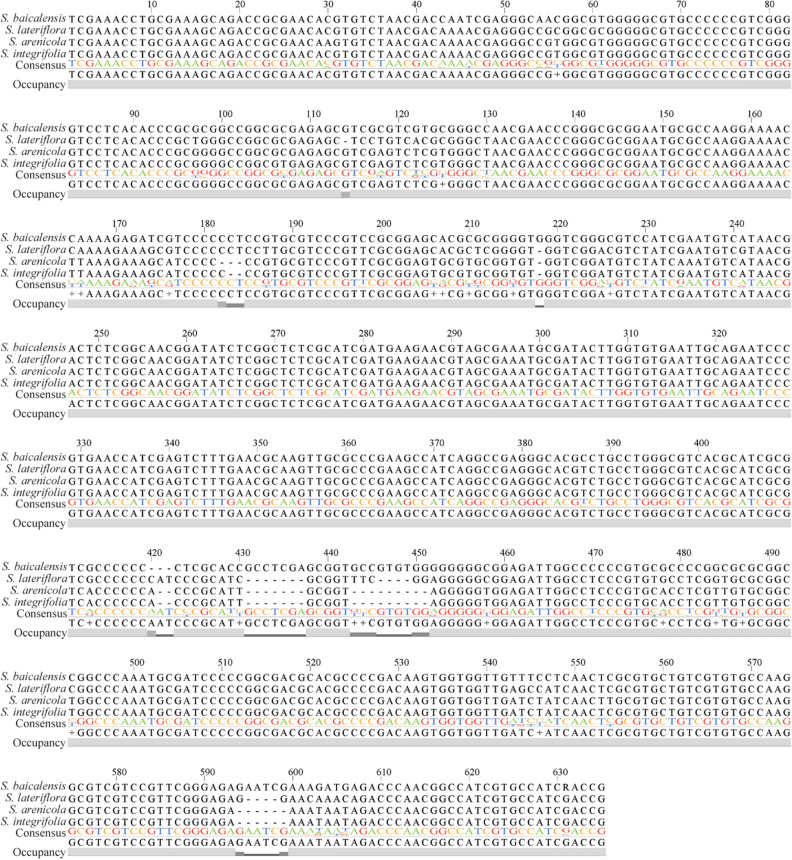
DNA barcoding using ribosomal internal transcribed spacer (*ITS*) marker. The polymorphisms identified in the *ITS* sequence between *Scutellaria baicalensis, S. lateriflora, S. arenicola, and S. integrifolia* are represented in this multiple sequence alignment that may be used for species identification.

All four *Scutellaria* species in this study, *S. baicalensis*, *S. integrifolia*, *S. arenicola,* and *S. lateriflora*, were successfully distinguished by DNA barcoding using nuclear ribosomal internal transcribed spacer (*ITS*) marker for identification. The multiple sequence alignments and representative consensus sequence indicated the single nucleotide polymorphisms and indels differentiating the species (Fig. 2).

**Figure 3:**
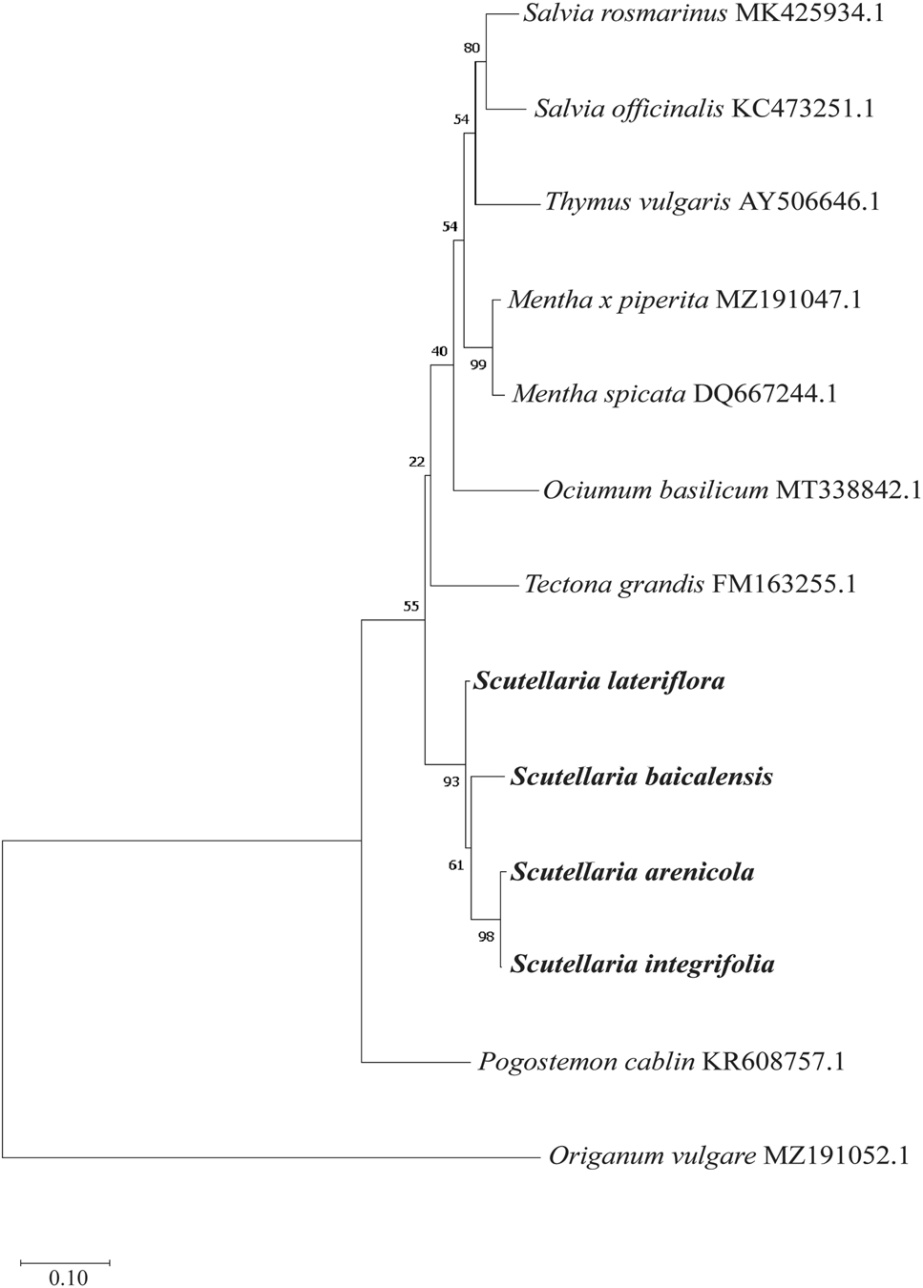
Phylogenetic analysis of selected Lamiaceae members using the *ITS* gene. A maximum-likelihood phylogenetic tree derived from multiple sequence alignments using the nuclear internal transcribed spacer (*ITS*) sequences of *Scutellaria* species with other Lamiaceae family members was constructed using MEGAv11.0 by maximum-likelihood (ML) method. Accessions in bold represent accessions sequenced for the current study. *Origanum vulgare* is included as an outgroup. Bootstrap values are from 1000 replicates, indicated above the nodes.

The length of the *ITS* sequences analyzed was 635 bp with a GC content of 59 – 63% and is composed of 103 polymorphic sites, including 18 indels detected between the species. The three native species could be easily distinguished from *S. baicalensis* due to InDels at multiple sites. *S. arenicola* and *S. integrifolia* are differentiated by only four SNPs at positions 30, 107, 234, and 554, along with a single base pair deletion at position 182 in *S. arenicola* (Fig. 2). To further evaluate the ability to discriminate species and establish phylogenetic relationships with other economically important species belonging to the Lamiaceae family, a phylogenetic analysis was performed based on *ITS* sequences from this study along with those deposited in the GenBank (Fig. 3). The average supporting values of nodes on each branch were mostly over 50%, indicating reliable evolutionary relationships. All the *Scutellaria* species were clustered together and separated from the outgroup *Origanum* species. Within *Scutellaria, S. arenicola* and *S. integrifolia* are clustered in the same branch with a maximum likelihood of 98, indicating they are genetically close species.

### Nuclear DNA content determination and ploidy estimation of *Scutellaria* species

The nuclear DNA content of all four *Scutellaria* species used in the study was determined using tomato as an internal reference (Table 1, Fig S1). *S. lateriflora* contained the highest content of DNA, almost double the amount compared to *S. baicalensis*. Interestingly, all three Florida native species, *S. arenicola*, *S. integrifolia,* and *S. lateriflora*, have a comparable amount of DNA, suggesting polyploidization.

**Table 1:** Nuclear DNA content of *Scutellaria* species in this study.

Species	Nuclear DNA Content ± SE (pg/2C)
*S. baicalensis*	0.94 ± 0.01 c
*S. lateriflora*	1.58 ± 0.01 a
*S. arenicola*	1.55 ± 0.03 a
*S. integrifolia*	1.43 ± 0.01 b

Data are presented as mean ± Standard error (SE). Means with different letters are significantly different (Tukey HSD test, p < 0.05).

### Differential accumulation of flavones in aerial and underground tissues revealed by comparative metabolite profiling

**Figure 4:**
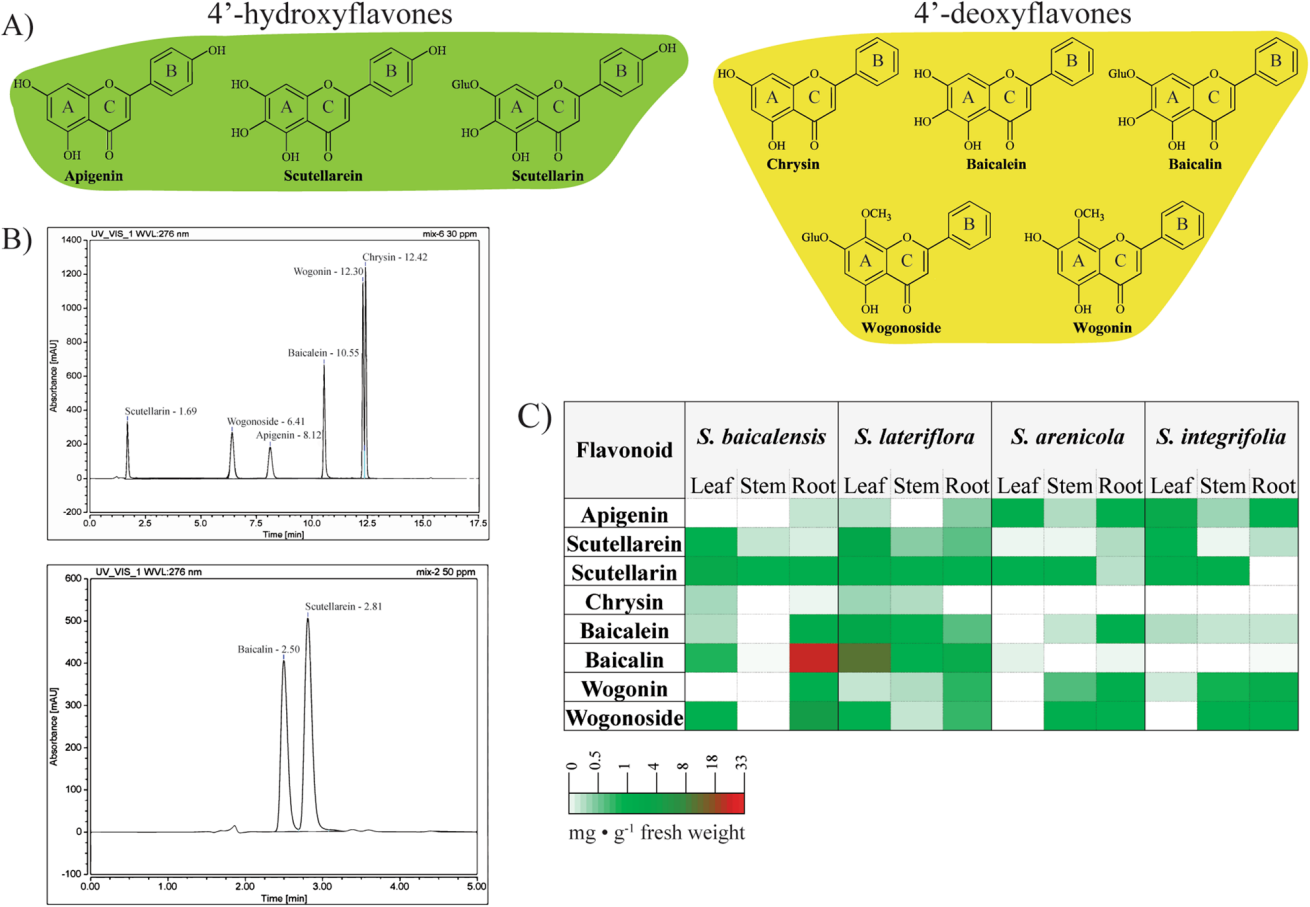
Identification and quantification of selected flavones from four *Scutellaria* species used in this study. (**A**) Structures of the 4′—hydroxyflavones and 4′ -deoxyflavones analyzed in the study. (**B**) External standard chromatograms obtained by HPLC analysis. (**C**) Heat map, indicating relative concentrations of all eight flavones determined by HPLC along with their localization patterns in aerial and underground tissues of the *Scutellaria* species*.* HPLC = High performance liquid chromatography.

The flavone profiles of leaf, stem, and root tissues from four *Scutellaria* species, natives *S. arenicola*, *S. lateriflora,* and *S. integrifolia*, and well-studied *S. baicalensis* were analyzed by HPLC. Three 4′-hydroxyflavones including apigenin, scutellarein, and its glucoside scutellarin, and five 4′-deoxyflavones that lack the 4′-OH group on the B ring, including chrysin, baicalein, and its glucoside baicalin, wogonin, and its glucoside wogonoside, were identified using external standards (Fig. 4A,B) and quantified based on the standard curves (Fig S2) derived by injecting varied volumes of known standard concentrations on the HPLC. Flavonoid profiles of all four *Scutellaria* species varied in composition and localization (Fig. 4C, Table 2). All species were found to contain all eight flavones investigated within this study, apart from chrysin not being detected in either *S. arenicola* or *S. integrifolia.* In *S. baicalensis*, a well-studied medicinal plant used in TCM, 4′-hydroxyflavones accumulated preferentially in leaves, while 4′-deoxyflavones accumulated in the underground root tissues (Fig. 4C, Table 2). A similar trend was observed in other native species except for *S. lateriflora*, where most flavones accumulated in aerial tissues.

**Table 2:** *Scutellaria* species flavonoid profiles and localization within leaf, stem, and root tissues.

	Apigenin	Scutellarein	Scutellarin	Chrysin	Baicalein	Baicalin	Wogonin	Wogonoside
***S. baicalensis***								
Leaf	N.D	0.34 ± 0.1^b^	1.59 ± 0.6^a^	0.1 ± 0.0^a^	0.09 ± 0.0^ab^	0.24 ± 0.1^c^	0.01 ± 0.0^bc^	0.27 ± 0.0^b^
Stem	N.D	0.07 ± 0.0^b^	0.38 ± 0.0^bc^	N.D	N.D	0.02 ± 0.0^c^	N.D	N.D
Roots	0.07 ± 0.0^ab^	0.05 ± 0.0^b^	0.89 ± 0.1^abc^	0.03 ± 0.0^a^	1.27 ± 0.5^ab^	26.05 ± 3.9^a^	0.46 ± 0.1^b^	3.82 ± 0.9^a^
***S. lateriflora***								
Leaf	0.08 ± 0.0^b^	2.48 ± 0.4^a^	1.3 ± 0.2^ab^	0.11 ± 0.0^a^	2.24 ± 0.7^a^	11.66 ± 2.9^b^	0.07 ± 0.0^c^	0.44 ± 0.0^b^
Stem	N.D	0.13 ± 0.0^b^	0.52 ± 0.0^bc^	0.09 ± 0.0^a^	0.62 ± 0.1^b^	0.27 ± 0.1^c^	0.09 ± 0.0^c^	0.07 ± 0.0^b^
Roots	0.13 ± 0.0^b^	0.18 ± 0.0^b^	0.43 ± 0.1^bc^	N.D	0.35 ± 0.1^b^	1.48 ± 0.4^c^	0.23 ± 0.0^bc^	0.22 ± 0.0^b^
***S. arenicola***								
Leaf	0.85 ± 0.2^ab^	0.03 ± 0.0^b^	0.41 ± 0.0^bc^	N.D	N.D	0.04 ± 0.0^c^	N.D	N.D
Stem	0.09 ± 0.0^b^	0.03 ± 0.0^b^	0.58 ± 0.1^bc^	N.D	0.07 ± 0.0^b^	0.01 ± 0.0^c^	0.19 ± 0.0^bc^	0.51 ± 0.1^b^
Roots	0.59 ± 0.2^b^	0.09 ± 0.0^b^	0.08 ± 0.0^c^	N.D	0.51 ± 0.1^b^	0.03 ± 0.0^c^	0.91 ± 0.1^a^	0.39 ± 0.1^b^
***S. integrifolia***								
Leaf	1.55 ± 0.4^a^	0.44 ± 0.1^b^	0.86 ± 0.1^abc^	N.D	0.09 ± 0.0^b^	0.01 ± 0.0^c^	0.06 ± 0.0^c^	N.D
Stem	0.11 ± 0.0^b^	0.03 ± 0.0^b^	0.71 ± 0.2^abc^	N.D	0.07 ± 0.0^ab^	0.01 ± 0.0^c^	0.25 ± 0.1^bc^	0.5 ± 0.1^b^
Roots	0.29 ± 0.1^b^	0.08 ± 0.0^b^	N.D	N.D	0.07 ± 0.0^b^	0.02 ± 0.0^c^	1.03 ± 0.1^a^	0.29 ± 0.1^b^

Data are presented as means ± standard errors reported in mg g^−1^ of fresh weight. Means with different letters are significantly different (Tukey honestly significant difference (HSD), p < 0.05). Statistical analysis was performed for each metabolite across different species and tissue types. N.D. indicates levels below detectable thresholds.

Of the eight flavones quantified in this study, 4′-deoxyflavone baicalin is the most abundant flavone detected with relatively high concentrations in roots of *S. baicalensis* (26.1 ± 3.9 mg g^−1^) and leaves of *S. lateriflora* (11.7 ± 2.9 mg g^−1^) (Fig. 4C and Table 2). Baicalein concentration was relatively lower than its glycoside baicalin in *S. baicalensis* (1.3 ± 0.7 mg g^−1^). However, *S. lateriflora* leaf had the highest amount of baicalein (2.24 ± 0.7 mg g^−1^) followed by its stems (0.6 ± 0.1 mg g^−1^) compared to other species. A comparable amount was also observed in *S. arenicola* root (0.5 ± 0.1 mg g^−1^). Another abundant 4′-deoxyflavone in *S. baicalensis* root is wogonoside (3.8 ± 0.9 mg g^−1^), found in relatively smaller amounts in other species. While its aglycone wogonin was detected in the roots of all four species, the highest amounts were detected in roots of native species *S. integrifolia* (1.0 ± 0.1 mg g^−1^) followed by *S. arenicola* (0.9 ± 0.1 mg g^−1^)*.* However, the precursor for 4′-deoxyflavones, chrysin was not detected in most tissues except for insignificant amounts in aerial parts of *S. baicalensis* and *S. lateriflora.*

4′-hydroxyflavones, scutellarein, and scutellarin were relatively abundant in aerial tissues and were most widely detected flavones across all the species and tissue types investigated. Interestingly, *S. lateriflora* primarily accumulated both 4′-hydroxy and 4′-deoxyflavones in aerial leaf tissues and more abundantly than other species analyzed in this study. The precursor of 4′-hydroxyflavones, apigenin, was either not detected or found in insignificant amounts in *S. baicalensis* and *S. lateriflora*. The highest amounts of apigenin among the species and tissues analyzed in this study were detected in the leaf tissue of *S. integrifolia* (1.6 ± 0.4 mg g^−1^) followed by *S. arenicola* (0.9 ± 0.2 mg g^−1^). Overall, the localization pattern of flavones in native species, *S. arenicola* and *S. integrifolia,* is similar to *S. baicalensis,* where 4′-deoxyflavones accumulate in the root tissues, and 4′-hydroxyflavones primarily accumulate in aerial tissues, unlike *S. lateriflora* where aerial parts are rich in all flavones. For example, the sum of all eight flavones identified in leaves of *S. lateriflora* was 18.4 mg g^−1^ of fresh weight, while *S. baicalensis*, *S. arenicola,* and *S. integrifolia* were 2.6, 1.3, and 3.0 mg g^−1^, respectively.

The retention times of multiple significant peaks during the flavone analysis of these *Scutellaria* species by HPLC did not match the eight flavone standards used in this study especially in *S. arenicola* and *S. baicalensis*. We hypothesized that some of these dominant peaks in the profile correspond to other flavones unique to the species.

### LC–MS/MS characterization of unknown metabolites in *Scutellaria* species

**Figure 5:**
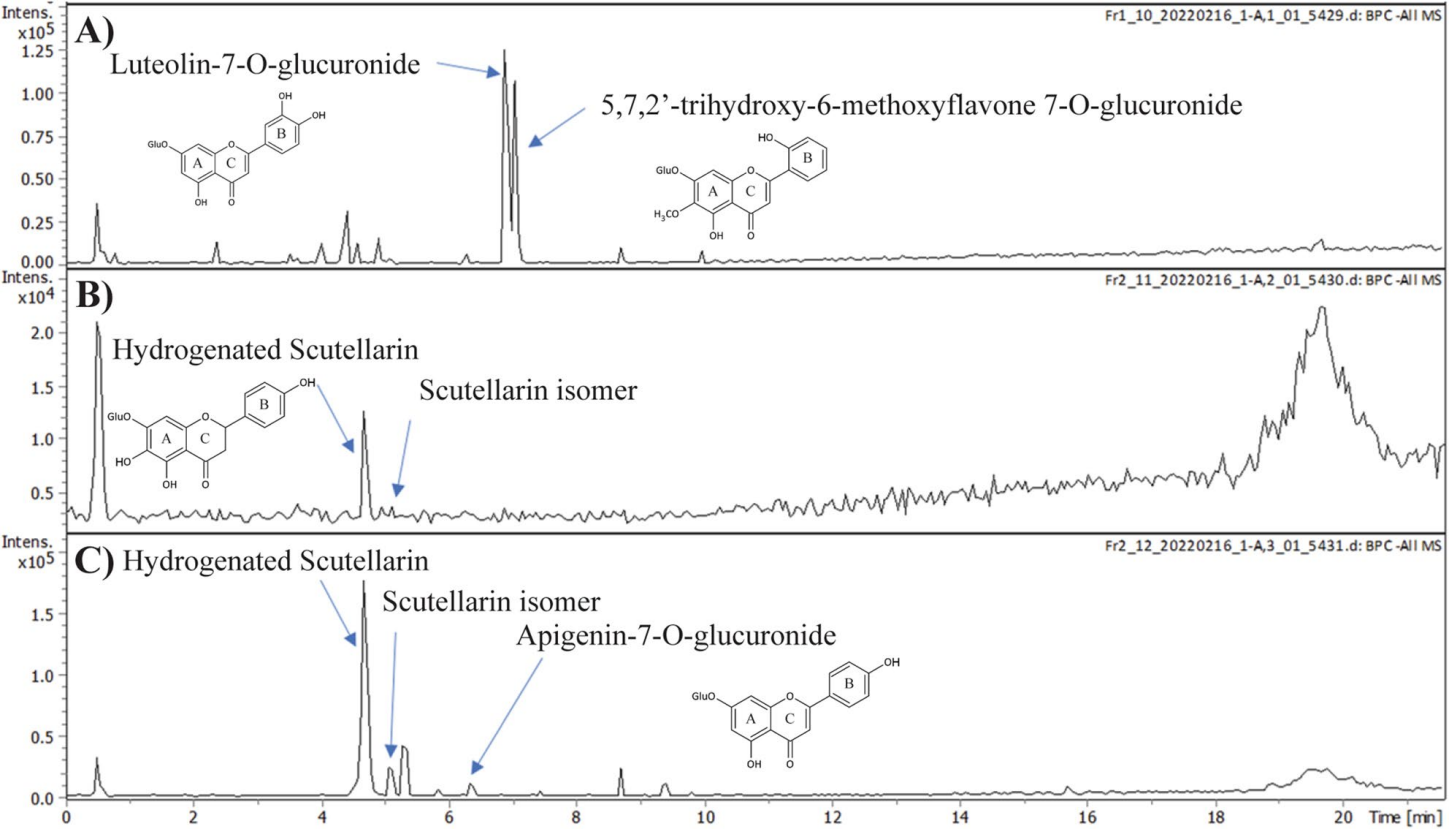
LC–MS/MS identification of the unknown peaks in the HPLC fractions (**A**) *S. arenicola* unknown fraction 1–10 with two identified metabolites and their chemical structures. *S. baicalensis* unknown fractions (**B**) 2–11 and (**C**) 2–12 showing identified metabolites and their chemical structures. Please refer to Supplemental Fig. S4-S6 for detailed MS1 and MS2 spectra supporting the level 2 identification of the metabolites.

In *S. baicalensis,* a significant peak coeluted shortly after the scutellarin standard while exhibiting a later retention time than the standard. In *S. arenicola,* two significant peaks were eluted at times significantly different from the flavone standards used for analysis. These unidentified peaks dominated the flavone profile, especially in *S. arenicola*. These unidentified metabolites were collected through HPLC fractionation, yielding one fraction for *S. arenicola* (1–10) and two fractions for *S. baicalensis* (2–11, 2–12) (Fig. 5). Using LC–MS/MS, unknown peaks in the three fractions 1–10, 2–11 and 2–12 were identified. The characterization of the unknown metabolites was performed using level 2 identification. It includes MS1, MS2, structural fragmentation along with spectral match to databases and libraries. With the authentic scutellarin standard, MS1 and MS2 spectra were also acquired. Please note that the purchased scutellarin standard had traces of apigenin-7-O-glucuronide and diosmetin (Fig S3). Fraction 1–10 from *S. arenicola* has two major peaks, which were identified to be luteolin-7-O-glucuronide and 5,7,2′-trihydroxy-6-methoxyflavone 7-O-glucuronide (Fig. 5A, S4). The identification was based on accurate mass of the precursors (461.0804 and 475.0963, respectively) and their corresponding fragments in the MS/MS spectra (Fig S4). Scutellarin standard assisted the potential identification of scutellarin or isomers in fractions 2–11 and 2–12 from *S. baicalensis* (Fig. 5B,C, S5, S6). Fraction 2–11 had a major peak which also appeared in fraction 2–12 at retention time 4.63 min. Its MS/MS spectrum is very similar to scutellarin's (*m/z* 461.0726). The [M–H]^−^ = 463.0893, 2 mass units more than scutellarin. Its major fragments are also two more than scutellarin, suggesting that it has one bond fewer than scutellarin. We thus propose this unknown to be hydrogenated scutellarin (Fig S5, S6). Scutellarin checkmarks all the level 2 identification criteria and therefore the likelihood of unknown to be hydrogenated scutellarin is high. In Fraction 2–12, the major peak was hydrogenated scutellarin, apparently in both dimeric and monomeric forms (Fig S5). The two other minor peaks were identified to be scutellarin isomer and apigenin-7-O-glucuronide (Fig S6).

## Discussion

Four species of *Scutellaria* have been profiled in this study, of which *S. baicalensis* is a well-known herb used in TCM, while the other three species are native to the Americas. There are significant differences between the species in plant architecture, flower morphology, and organ-specific localization of flavones (Figs. 1, 4, and Table 2). Contrary to the other species included in this study, *S. lateriflora* produces small blue flowers, ~ 1 cm long, and has a high accumulation of flavones in the aerial tissues. Other species exhibit showy flowers with flavones accumulated in underground roots. These variations may be attributed to diverse habitats and evolutionary adaptation to these habitats. For example, *S. arenicola* thrives in a dry habitat while *S. lateriflora* grows in a wetland habitat^24,25^. *ITS* is a widely used DNA barcoding marker for photosynthetic eukaryotic organisms^26^. *ITS* sequence data is deposited from various species in the GenBank, and it is commonly used for phylogeny construction and performed comprehensively in *Scutellaria* species^27^. DNA barcoding and phylogenetic analyses performed in this study indicated that natives, *S. arenicola,* and *S. integrifolia*, are phylogenetically closely related species and had several polymorphic InDels and SNPs compared to *S. baicalensis* and *S. lateriflora*. Furthermore, the total DNA content of *S. baicalensis* measured was 0.94 pg/2C, while *S. lateriflora* was 1.58, congruent with previous reports^27–29^. Ploidy of *Scutellaria* species ranges from diploid to octoploid and chromosome counts established that *S. baicalensis* is a diploid, while *S. lateriflora* is a tetraploid species^19,20,30,31^. In this study, the DNA content measured from *S. arenicola* and *S. integrifolia* is similar to *S. lateriflora*, suggesting that the other two native species may be tetraploid as well.

Flavones have a variety of biological roles in plants including acting as co-pigments with anthocyanins, Ultraviolet B protectants in plants, and protection against insects and fungal pathogens^32–34^. The first step in the formation of 4′-hydroxyflavones is a dehydration reaction of flavanone naringenin catalyzed by a flavone synthase (SbFNSII-1) to form apigenin, a precursor for 4′-hydroxyflavones. However, the 4′-deoxyflavones are produced from chrysin formed by dehydration of flavanone pinocembrin catalyzed by SbFNSII-2^3,23^. FNS is restricted to various land plant species that synthesize flavones including basal liverworts^35–38^, and various forms of FNS can also be found in these species, including cytochrome P450, 2-oxoglutarate-dependant dioxygenase, etc.,^39^. In this study, we see a differential accumulation of flavones in aerial and root tissues of *Scutellaria*, suggesting the functional plasticity of FNS as an evolutionary adaptation to environmental variations. Interestingly, when scutellarein and scutellarin were accumulated in higher amounts, lower precursor compound apigenin was detected, as observed in *S. baicalensis* and *S. lateriflora*. Aerial tissue of *S. baicalensis* was also found to contain relatively high amounts of hydrogenated scutellarin, along with a minor amount of a scutellarin isomer and apigenin-7-O-glucuronide (Fig. 5). A relatively high amount of apigenin was detected in the leaves of natives, *S. integrifolia,* and *S. arenicola*, suggesting differential hydroxylation of apigenin resulting in a lower concentration of downstream 4′-hydroxyflavones.

The flavone profile of *S. arenicola* leaf tissues also significantly differentiated this species from the others included in this study. LC–MS/MS identified the major unknown peaks from the HPLC analysis to be luteolin-7-O-glucuronide and 5,7,2′-trihydroxy-6-methoxyflavone 7-O-glucuronide (Fig. 5 and Fig S4). These peaks were not observed in as significant concentrations in other species. Both luteolin and its glycoside, similar to other flavones included in this study, have exhibited anti-inflammatory activity in both in vitro and animal models^40–43^. Luteolin has been shown to beneficially modulate neurotrophic signaling pathways, resulting in the protection or growth of neurons^43^. The predominant accumulation of luteolin-7-O-glucuronide in *S. arenicola*, implores further investigation of this species for its medicinal properties. While the biological significance of 5,7,2′-trihydroxy-6-methoxyflavone 7-O-glucuronide which was previously detected in relatively minor quantities in *S. baicalensis* root^44–46^ is still unclear, this compound was previously shown to have antioxidant activity^47^.This warrants further investigation into the potential bioactivity of this metabolite which is accumulated abundantly in *S. arenicola*. The detection of these dominant metabolites in the leaf tissues of *S. arenicola* further supports the differential accumulation of flavones in aerial and root tissues suggesting diverse physiological roles of these metabolites in plants.

Historically, Baikal roots have been used in TCM^18^, while native Americans used aerial tissues of *S. lateriflora* as ethnobotanical sources^48^. This is consistent with findings of others and in our study that bioactive flavones are differentially deposited in these species. From this study, *S. arenicola* and *S. integrifolia* are closely related species with similar patterns of flavone profiles and distribution within the plant tissues. Furthermore, wogonin and wogonoside are in higher concentrations in these two species than the other two, which have high baicalin and baicalein. Although chrysin is the precursor compound for 4′-deoxyflavones, the hydroxylation of chrysin is catalyzed by flavone-6-hydroxylase to yield baicalein and baicalin, whereas flavone-8-hydroxylase activity results in wogonin and wogonoside^3,23^. Preferential expression of either enzyme may result in the corresponding differences in the chemical profile. Chrysin, by itself, was either detected in relatively low amounts in *S. baicalensis* and *S. lateriflora*, while no chrysin was detected in the tissues of *S. arenicola* and *S. integrifolia.* These findings suggest that chrysin gets metabolized into downstream 4′-deoxyflavones in all species analyzed in this study.

Various commercial products are available for *S. baicalensis* and *S. lateriflora*, which are easily accessible through online or retail sources*.* Both species are currently listed in both American and Chinese pharmacopeias^49,50^. Given the high potential of *Scutellaria* species to serve as both a popular ornamental landscape plant and an important source of natural product medicine, research is needed to identify the best germplasm with the highest amounts of bioactive metabolites while evaluating the species under controlled environments to maximize the biosynthesis of these pharmaceuticals.

## Methods

### Plant material and growth conditions

Seeds of *S. baicalensis, S. lateriflora* and *S. arenicola* were obtained from Floral Encounters (https://www.floralencounters.com), Strictly Medicinal Seeds (https://strictlymedicinalseeds.com/) and Dr. Jeongim Kim at the University of Florida respectively. *S. integrifolia* plants were purchased from Wood Thrush Native Nursery (Floyd, VA, U.S.). Plants started from seeds and cuttings were grown in 16.5 cm containers in PRO-MIX BX (Premier Tech Horticulture, Quakertown, PA, U.S.) supplemented with Osmocote 18–6-12 control release fertilizer (Scotts, Marysville, OH, U.S.) at the labeled medium rate of 24 g·gallon^−1^ in a greenhouse under a light intensity of 650 μmol m^−2^ s^−1^ and average temperature of 24 °C.

### DNA barcoding of *Scutellaria* germplasm

Genomic DNA was extracted from young immature leaves of *Scutellaria* species using hexadecyltrimethylammonium bromide (CTAB)-based method^51^. The *ITS* was amplified using mixed base oligos ITS-u1 GGAAGKARAAGTCGTAACAAGG and ITS-u4 RGTTTCTTTTCCTCCGCTTA^52^. All PCR reactions were performed using Phusion® High-Fidelity DNA Polymerase (New England BioLabs, Ipswich, MA, U.S.). The PCR products were purified using the Wizard® SV Gel and PCR Clean-Up System (Promega Madison, WI, U.S.) and sequenced. A-tails were added to the purified PCR products and ligated into pGEM®-T Easy Vector System I (Promega, Madison, WI, U.S.). Colonies were screened for *ITS* using SP6 ATTTAGGTGACACTATAG and T7 TAATACGACTCACTATAGGG primers. Positive colonies were cultured in LB liquid media with ampicillin (100 mg·ml^−1^) selection and plasmids isolated using the Wizard® Plus SV Minipreps DNA Purification System (Promega, Madison, WI, U.S.). Sanger sequencing of the plasmids (1000 ng) and purified PCR products (40 ng) was performed at GeneWiz (https://www.genewiz.com/) using SP6 and ITS-U1 primers, respectively. Sequencing reads were analyzed by Sequencher version 5.4.6 (Gene Codes Corp., Ann Arbor, MI, U.S.).

### Molecular phylogenetic analysis

The *ITS* sequences were aligned, and analyses of pairwise genetic distances were computed with the Kimura-2-parameter (K2P) model using MUSCLE in MEGA11. A maximum likelihood phylogenetic tree was generated using multiple sequence alignment of the DNA sequences of selected species using MEGA11.

### Determination of nuclear DNA content by flow cytometry

Nuclear DNA content of *Scutellaria* species was determined using an Accuri C6 flow cytometer (BD Biosciences, San Jose, CA, U.S.) using tomato (*Solanum lycopersicum* L.' Stupické polní rané') as an internal standard^53^. The nuclear lysis buffer LB01 consisting of 15 mM Tris, 2 mM Na_2_EDTA, 0.5 mM spermine tetrahydrochloride, 80 mM KCl, 20 mM NaCl, 0.1% (vol/vol) Triton X-100 was selected for nuclear isolation after adjusting to 7.5 pH with 1 M NaOH. It is filtered with a 0.22-µm filter, and 15 mM β-mercaptoethanol is added to it. 30 mg of young leaves of *Scutellaria sp.* and tomato internal standard were finely cut and directly added to a petri dish containing one ml of LB01 buffer*.*. The homogenate was filtered through a nylon mesh (50 µm) and 50 µl of the DNA fluorochrome, propidium iodide (Sigma-Aldrich, St. Louis, MO, U.S.; 1 mg·ml^−1^) and RNase (Sigma-Aldrich, St. Louis, MO, U.S.; 1 mg·ml^−1^) were added to the filtered homogenate^54^. Three biological replicates of the two unreported species (*S. arenicola* and *S. integrifolia*) and a plant each for *S. baicalensis* and *S. lateriflora* were analyzed. The nuclear DNA content of each sample was calculated as nuclear DNA content of internal standard (('Stupické polní rané' tomato) × mean fluorescence value of sample ÷ Mean fluorescence value of internal standard)^53^.

### Extraction and identification of flavonoids by HPLC

Five biological replicates of leaf, stem, and root tissue from *Scutellaria* species were harvested two months after germination. Samples were flash-frozen in liquid-N_2_ and the plant tissues were ground to a fine powder using mortar and pestles with liquid-N_2_ and stored at -80˚C until extractions were performed. Thirty milligrams of ground tissue were combined with 1 ml of 80% HPLC grade MeOH (30,000 ppm). Samples were sonicated in an ultrasonic water bath at room temperature for 1.5 h and then centrifuged at 15,000 rpm at 4 °C for 5 min. The remaining supernatant was filtered using a 0.2 µm filter. Samples were then diluted to 5,000 ppm, and 200 µl of each sample was aliquoted into an HPLC vial for analysis according to^3^.

Scutellarin, baicalin, wogonoside, wogonin, and apigenin standards were obtained from Biosynth Carbosynth (Newbury, U.K.). Scutellarin, baicalein, and chrysin standards were ordered from Millipore Sigma (Burlington, MA, U.S.). The standard stock solutions were prepared by dissolving the compounds into DMSO (except baicalin) and then made up to volume using HPLC grade MeOH. 50 ppm (scutellarein and baicalin) and 30 ppm (scutellarin, wogonoside, apigenin, baicalein, wogonin, and chrysin) standard mixtures were used for identification and quantification of the metabolites.

The flavone extracts were analyzed using a Thermo Scientific UltiMate 3000 (Waltham, MA, U.S.) HPLC system. Ten microliters of sample were injected onto a 3 × 100 mm Acclaim RSLC 120 C18 column for reverse-phase separation. Two different methods were used to separate all the metabolites for quantification. Scutellarin, wogonoside, wogonin, apigenin, chrysin, and baicalein metabolites were separated by setting the column temperature to 40 °C. The flow rate was set to 0.5 mL/min and sample was eluted by a mixture of 0.1% formic acid (solvent A) and 100% acetonitrile (solvent B) with the following gradient, 0 to 6 min, 25% B; 6–9 min, 25–50% B, 9 to 11 min, 50% B; 11–16 min, 50–95% B; 16–20 min, 95% B; 20–21 min, 95–25% B; 21–24 min, 25% B (modified from^23^). Scutellarein and baicalin were eluted by a mixture of 0.5% formic acid (solvent A) and 100% acetonitrile (solvent B) with the following gradient: 0 to 2 min, 30% B; 2- 6 min, 30–60% B; 6–11 min, 60–85% B; 11 to 11.25 min, 85–99% B; 11.25–12.25 min, 99% B; 12.25–12.50 min, 99–30% B; 12.5–16 min, 30% B. A flow rate of 0.4 mL/min was used, and the column oven temperature was set to 30˚C^55^. Injection volumes of 8.0, 1.0, and 0.2 µl of 30 ppm standard mixes and 8.0, 1.0, and 0.1 µl of 50 ppm standard mixes were used to generate a standard curve for quantifying the metabolite concentration.

### Identification of flavonoid metabolites by LC–MS/MS

Five extracts each at 30,000 ppm were combined for both *S. arenicola* and *S. baicalensis* leaf tissues, lyophilized and resolubilized in 840 µl 80% MeOH, and injected into HPLC at 100 µl injection volume until there was no sample remaining. Samples were eluted by a mixture of 0.1% formic acid (solvent A) and 100% acetonitrile (solvent B) with the following gradient: 0 to 2 min, 25% B; 2- 6 min, 25% B; 6–9 min, 25–50% B; 11 to 15 min, 50–95 B%; 15–23 min, 95% B; 23–24 min, 95–5% B; 24–34 min, 5–5% B. A flow rate of 0.5 mL/min was used, and the column oven temperature was set to 25˚C. One isolated fraction from *S. arenicola* (1–10) and two isolated fractions from *S. baicalensis* (2–11 and 2–12) were used for identification by LC–MS/MS.

MS1 and MS2 analyses were carried out using the scutellarin standard and unknown sample fraction—*S. arenicola* unknown (1–10), *S. baicalensis* unknown 1 (2–11), and *S. baicalensis* unknown 2 (2–12). The initial sample was collected in methanol after fractionation with a concentration of 30,000 ppm. These samples were lyophilized in a vacuum concentrator till dryness, then resolubilized in 0.1% formic acid in water. LC–MS/MS analysis was performed on a maXis impact quadrupole-time-of-flight mass spectrometer (Bruker corporation, Billerica, MA, US) coupled to a ACQUITY UPLC (ultrahigh performance liquid chromatography) system (Waters corporation, Milford, MA, US). Separation was achieved on a C18 column (2.1 × 150 mm, BEH C18 column with 1.7-µm particles) (Waters corporation, Milford, MA, US) using a linear gradient and mobile phase A (0.1% formic acid) and B (B: acetonitrile). Gradient condition: B increased from 5 to 70% over 30 min, and then to 95% over 3 min, held at 95% for 3 min, then returned to 5% for equilibrium. The flow rate was 0.56 mL/min, and the column temperature was 60˚C.

Mass spectrometry was performed in the negative electrospray ionization mode with the nebulization gas pressure at 44 psi, dry gas of 12 L/min, dry temperature of 250˚C, and a capillary voltage of 4500 V. Mass spectral data were collected from 100 and 1500 m*/z*, and tandem mass spectrometry (MS/MS) data were acquired using Auto-MS/MS mode with collision energy (CE) from 10 to 60 eV depending on *m/z* of the ions. The number of precursors was set to 3, with a smart exclusion of 5 and active exclusion of 3 spectra. The MS and MS/MS data were auto- calibrated using sodium formate that was introduced into the end of the gradient after data acquisition. The raw data were visualized using a Bruker data analysis software and searched against publicly available metabolite spectral databases in the Bruker Metaboscape software version 2021 (Bruker corporation, Billerica, MA, US). The fragmentation in the MS/MS spectra has been deciphered using the Mass Frontier 8.0 SR1 software.

### Statistical analysis

Data are presented as means and standard errors unless stated otherwise. Statistical analyses were performed using Tukey–Kramer honestly significant difference test (P ≤ 0.05) in JMP Pro 15.0.0 (SAS Institute, Cary, NC).

All the methods including experimental research on plants and metabolite extractions were carried out in accordance with relevant national/international/legislative and institutional guidelines and regulations.

## Supplementary Information

Supplementary Information.

## Data Availability

The datasets generated and/or analyzed during the current study are available in the https://doi.org/10.7910/DVN/BGDQQB repository. The LC–MS files are uploaded to MetaboLights under the accession ID MTBLS4504. The *ITS* sequences for *Scutellaria* species have been deposited in the GenBank, accession numbers ON890131-ON890136.
