# Supplementary material for: Exploring native Scutellaria species provides insight into differential accumulation of flavones with medicinal properties
Source: Sci Rep. 2022 Aug 1;12:13201. doi: 10.1038/s41598-022-17586-1 (PMC9343603; doi:10.1038/s41598-022-17586-1)

Fig S1. Flow cytometry representative peaks to determine the relative DNA content of the four *Scutellaria* species used in the study. M1 represents peak area under the marked range in red for each *Scutellaria* species. M2 represents peak area of tomato, used as an internal standard.

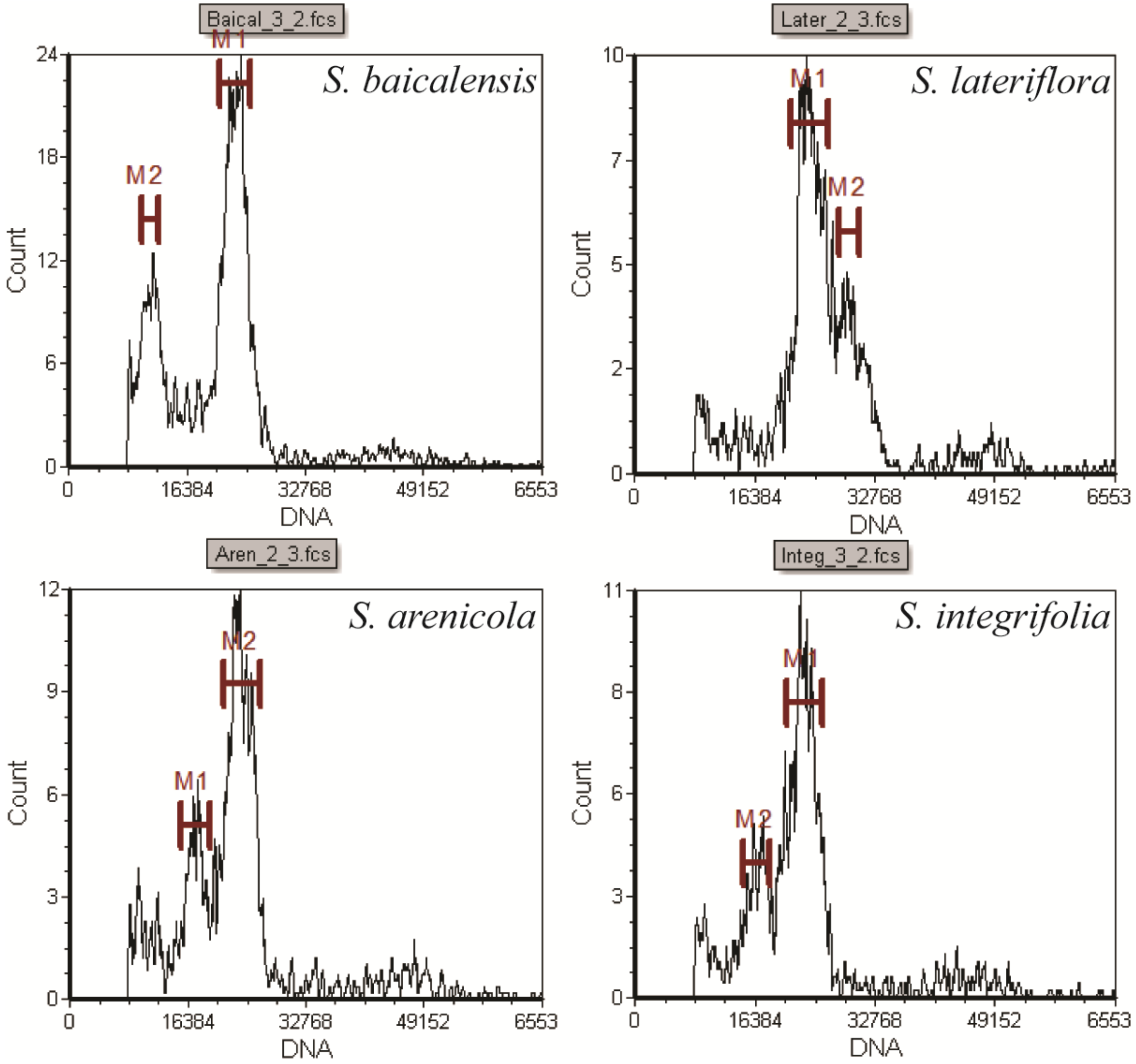

Fig S2. Representative HPLC standard curves for all the flavones identified and quantified in this study. Baicalin and scutellarein standard curves are auto-generated by HPLC using injection volumes of 8.0, 1.0, and 0.1 µl and R-Square:1.000. Apigenin, wogonoside, baicalein, wogonin, chrysin, and scutellarin standard curves are auto-generated with injection volumes of 8.0, 1.0, and 0.2 µl and R-Square:1.000.

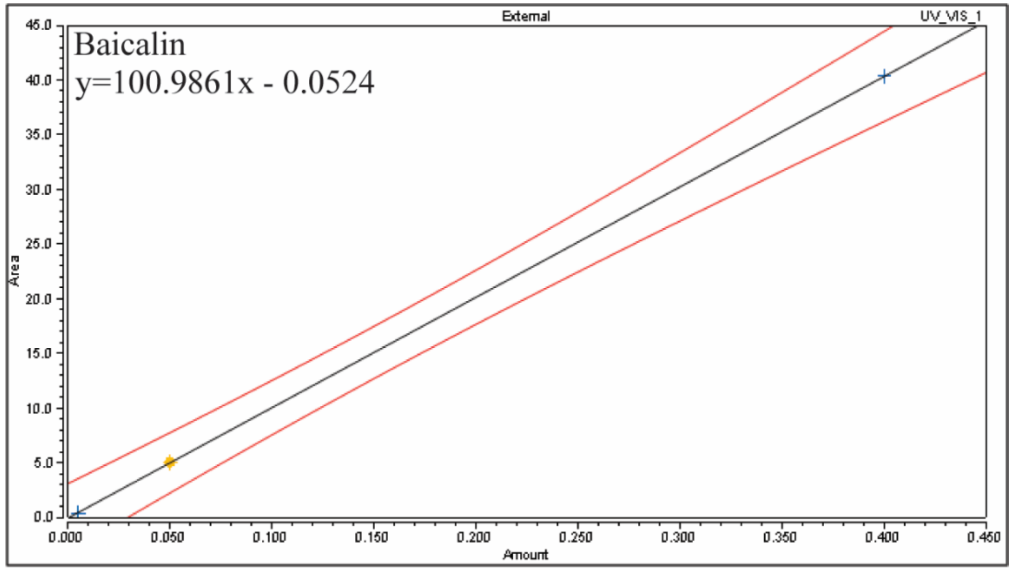

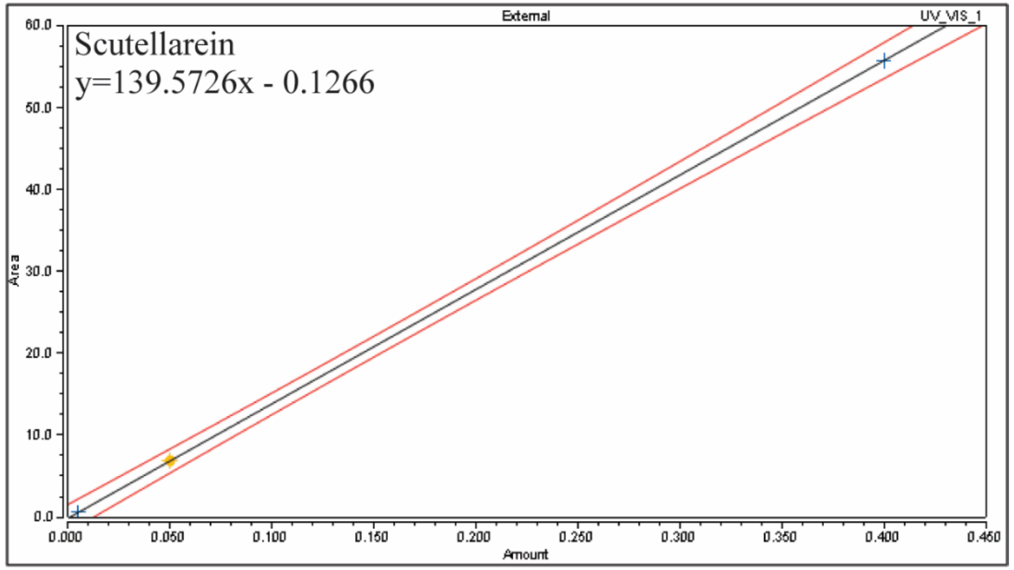

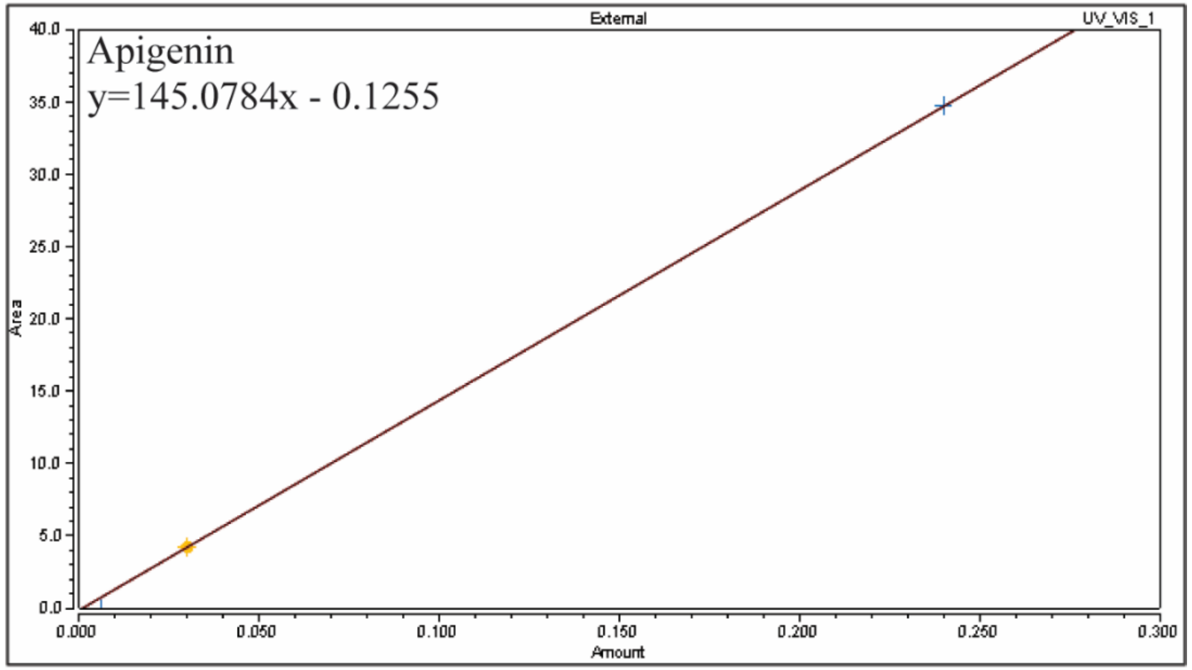
External
UV_VIS_1
Apigenin
y=145.0784x - 0.1255
Area
Amount
0.0
5.0
10.0
15.0
20.0
25.0
30.0
35.0
40.0
0.000
0.050
0.100
0.150
0.200
0.250
0.300

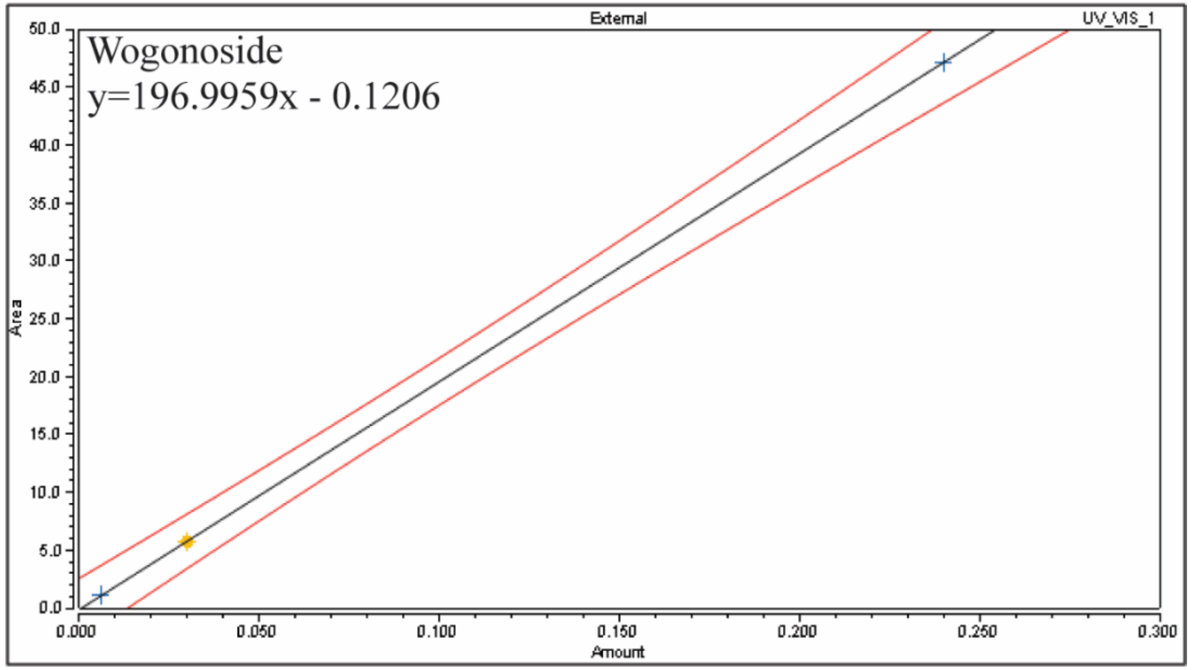
External
UV_VIS_1
Wogonoside
y=196.9959x - 0.1206
Area
Amount
0.0
5.0
10.0
15.0
20.0
25.0
30.0
35.0
40.0
45.0
50.0
0.000
0.050
0.100
0.150
0.200
0.250
0.300

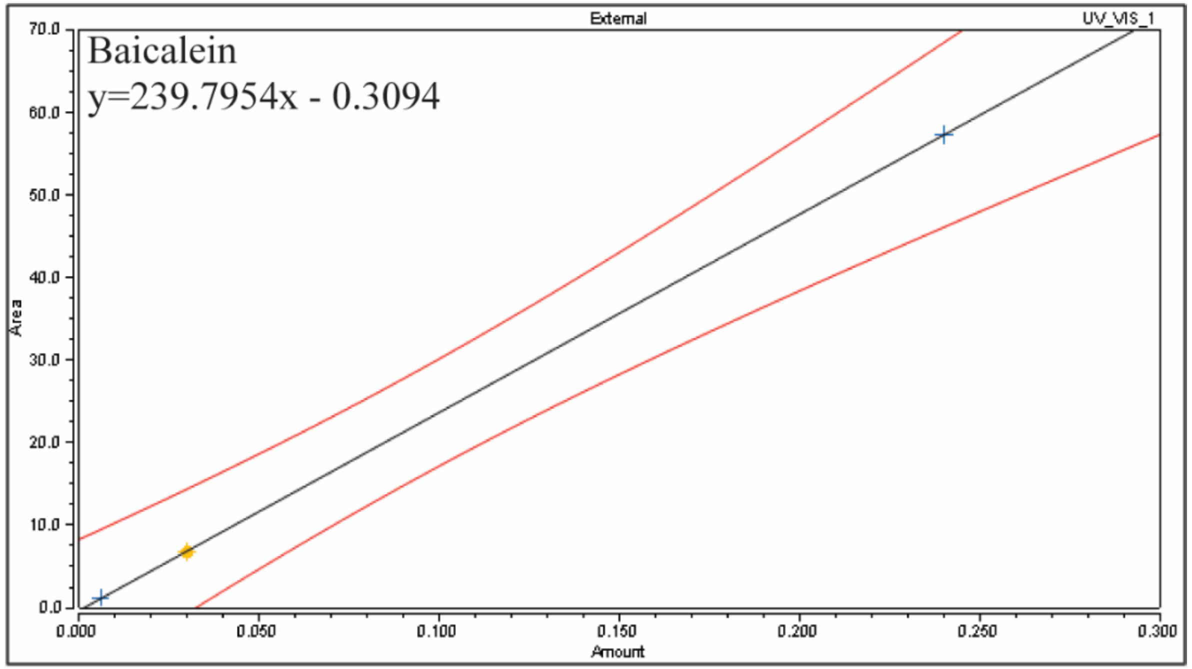
External
UV_VIS_1
Baicalein
y=239.7954x - 0.3094
Area
Amount
70.0
60.0
50.0
40.0
30.0
20.0
10.0
0.0
0.000
0.050
0.100
0.150
0.200
0.250
0.300

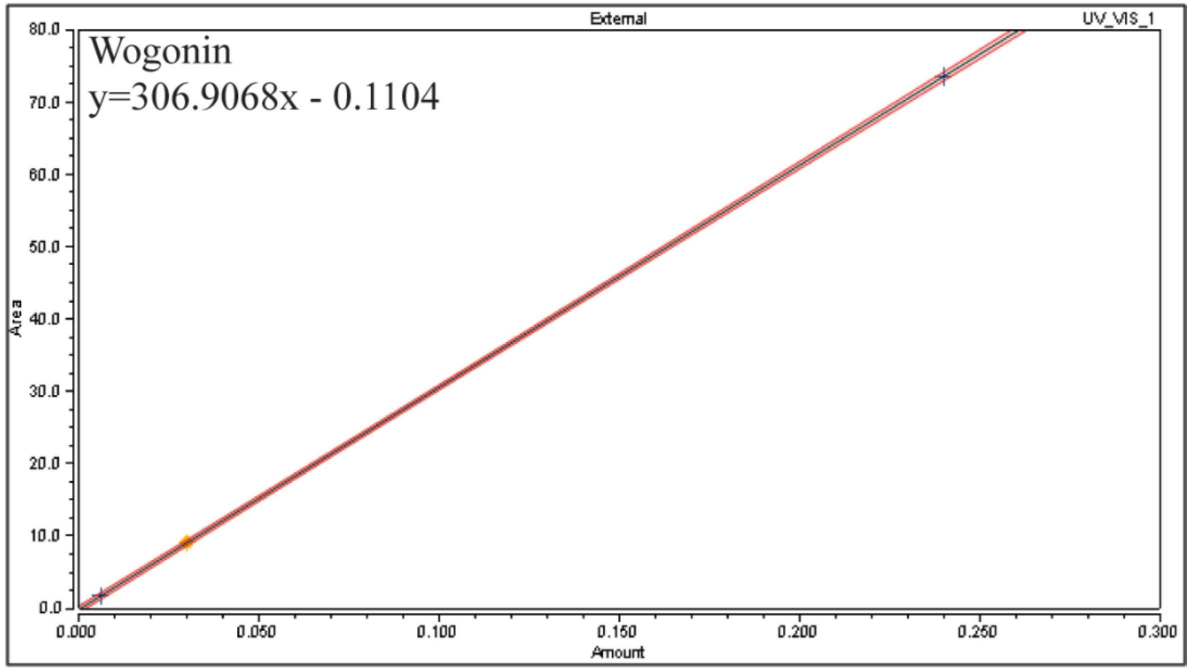
External
UV_VIS_1
Wogonin
y=306.9068x - 0.1104
Area
Amount
80.0
70.0
60.0
50.0
40.0
30.0
20.0
10.0
0.0
0.000
0.050
0.100
0.150
0.200
0.250
0.300

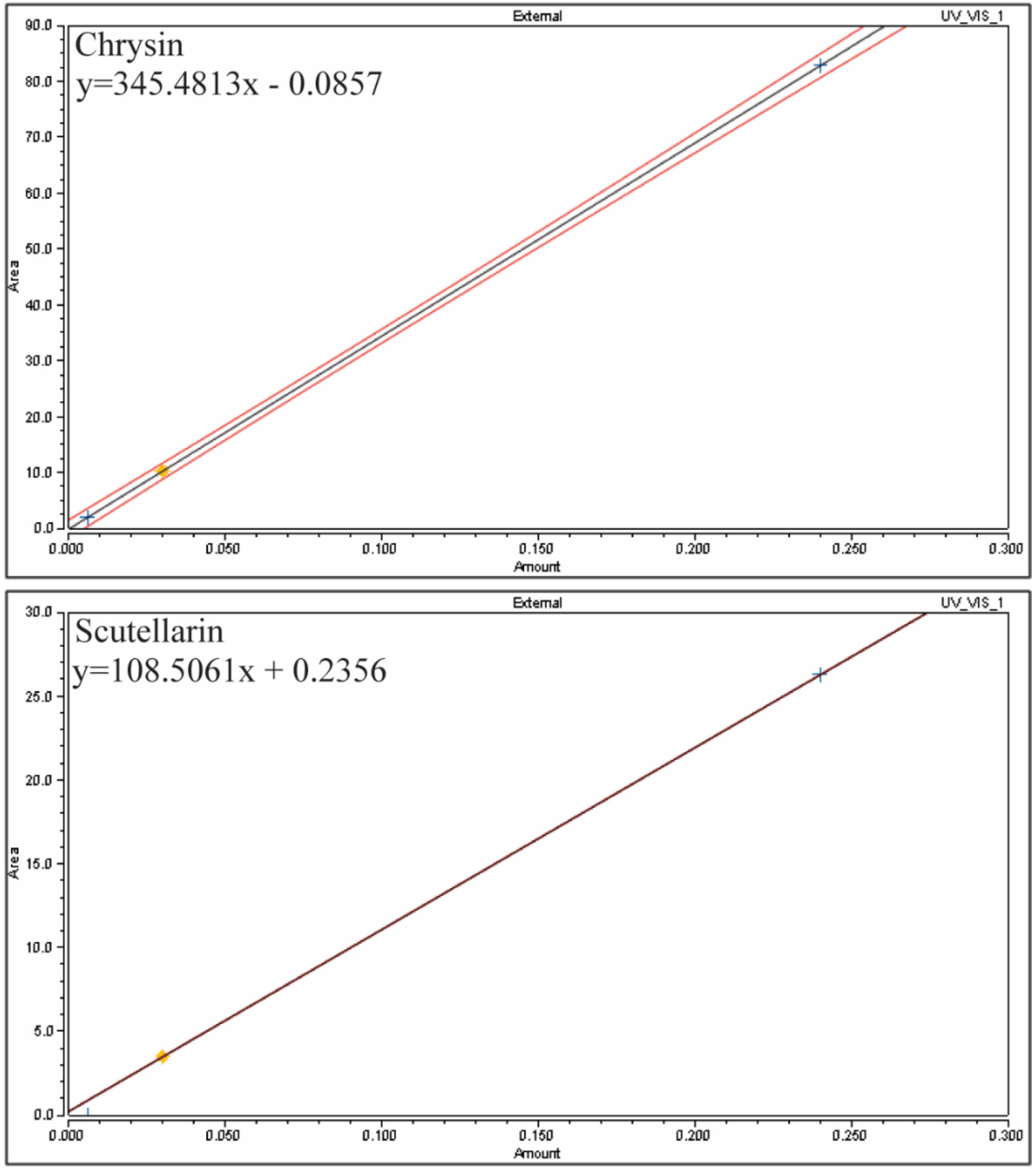
External
UV_VIS_1
Chrysin
y=345.4813x - 0.0857
Area
Amount
0.000
0.050
0.100
0.150
0.200
0.250
0.300
0.0
10.0
20.0
30.0
40.0
50.0
60.0
70.0
80.0
90.0
External
UV_VIS_1
Scutellarin
y=108.5061x + 0.2356
Area
Amount
0.0
5.0
10.0
15.0
20.0
25.0
30.0

Fig S3. LC-MS/MS data for Scutellarin standard used in the study. Scutellarin standard had small amounts of apigenin-7-O-glucuronide and diosmetin metabolites in it, confirmed using MS/MS.

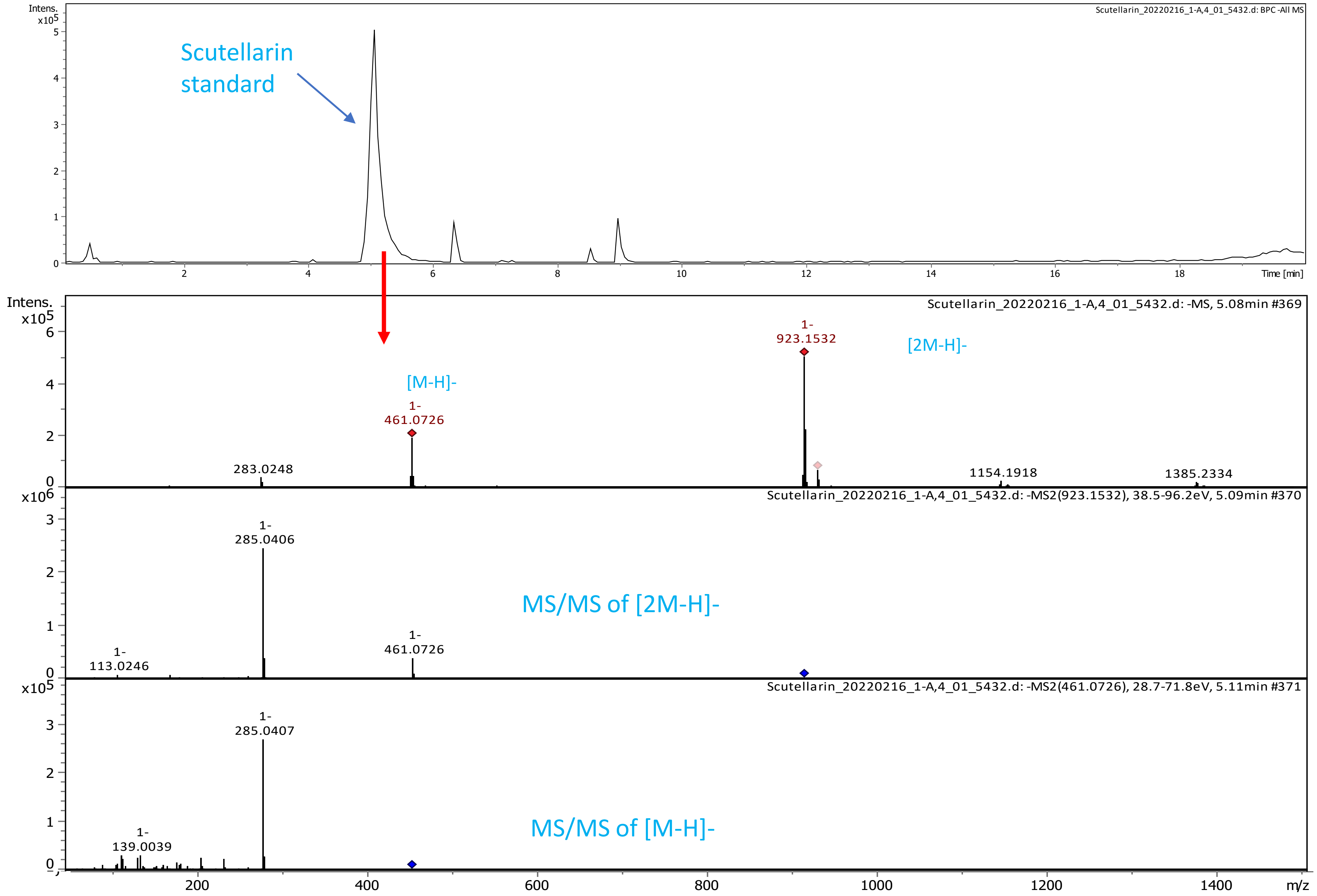

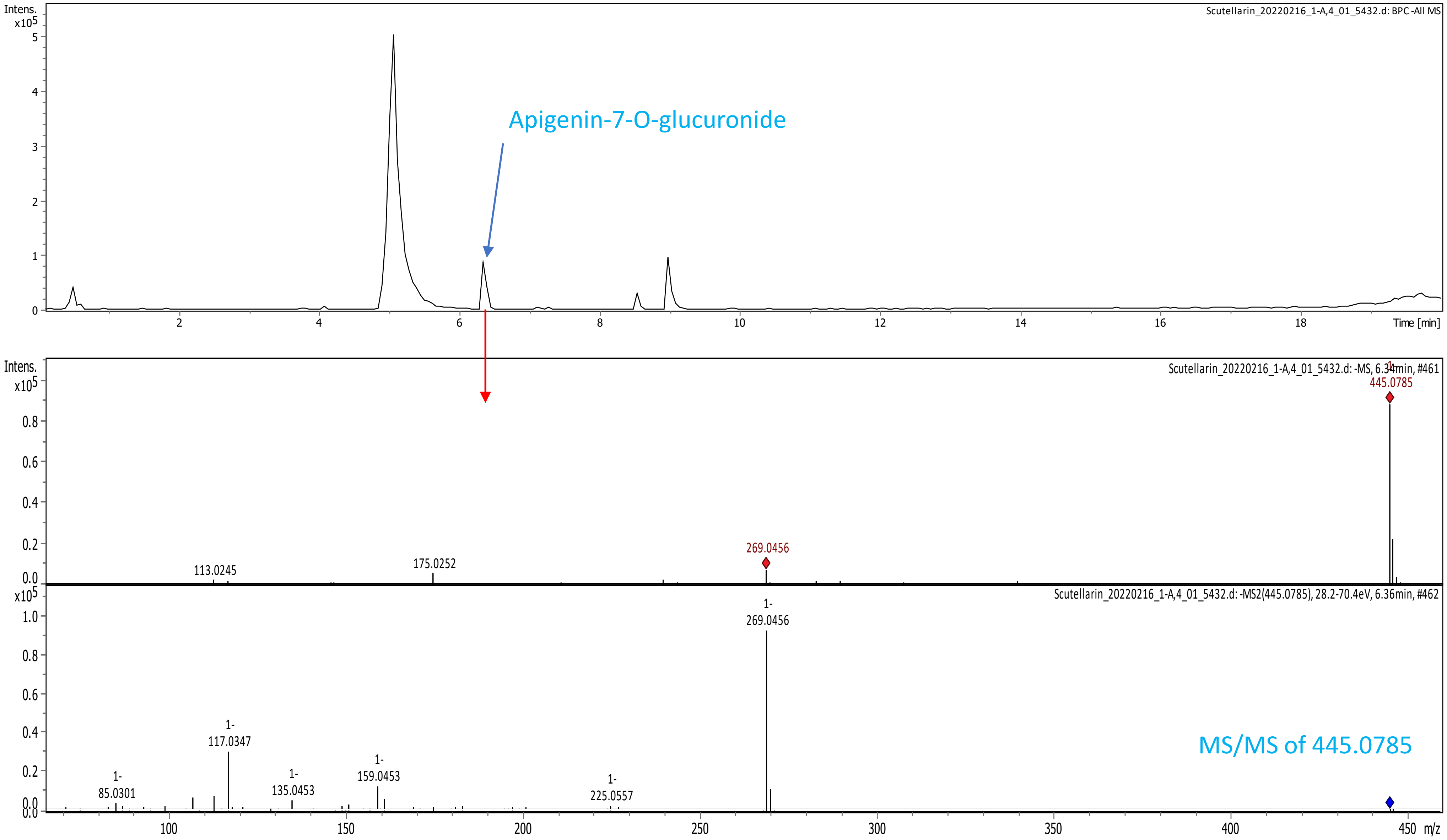
Apigenin-7-O-glucuronide
Scutellarin_20220216_1-A,4_01_5432.d: BPC -All MS
Intens.
x10^5
Time [min]
Scutellarin_20220216_1-A,4_01_5432.d: -MS, 6.34min, #461
445.0785
269.0456
175.0252
113.0245
Scutellarin_20220216_1-A,4_01_5432.d: -MS2(445.0785), 28.2-70.4eV, 6.36min, #462
1-
269.0456
1-
117.0347
1-
85.0301
1-
135.0453
1-
159.0453
1-
225.0557
MS/MS of 445.0785
m/z

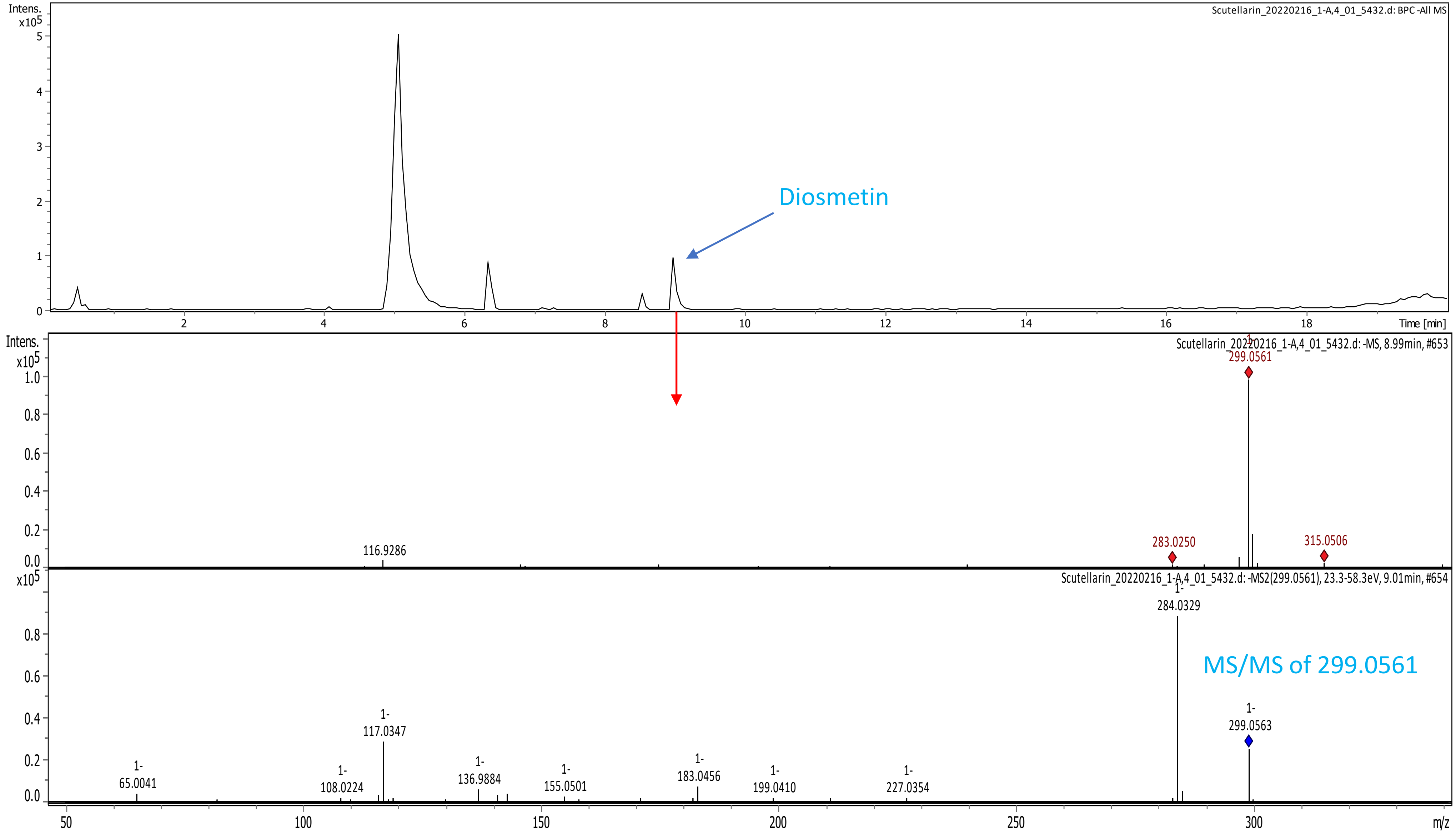
Intens.
x10^5
Scutellarin_20220216_1-A,4_01_5432.d: BPC -All MS
Diosmetin
Time [min]
Scutellarin_20220216_1-A,4_01_5432.d: -MS, 8.99min, #653
299.0561
283.0250
315.0506
116.9286
Scutellarin_20220216_1-A,4_01_5432.d: -MS2(299.0561), 23.3-58.3eV, 9.01min, #654
1- 284.0329
MS/MS of 299.0561
1- 299.0563
1- 117.0347
1- 65.0041
1- 108.0224
1- 136.9884
1- 155.0501
1- 183.0456
1- 199.0410
1- 227.0354
m/z

Fig S4. HPLC, Fractionation, and LC-MS/MS analysis for identification of *S. arenicola* unknown compounds in fraction 1-10. A) HPLC chromatogram of the whole extract and chromatogram of fraction collected from unknown major peaks in whole extract. 1, and 2 represent the metabolites identified whose MS/MS data is presented. B) MS1 spectrum of Luteolin-7-O-glucuronide showing accurate precursor mass and MS2 spectrum of the precursor C) MS1 spectrum of 5,7,2' – trihydroxy-6-methoxyflavone 7-O-glucuronide showing accurate precursor mass and MS2 spectrum of the precursor.

A)

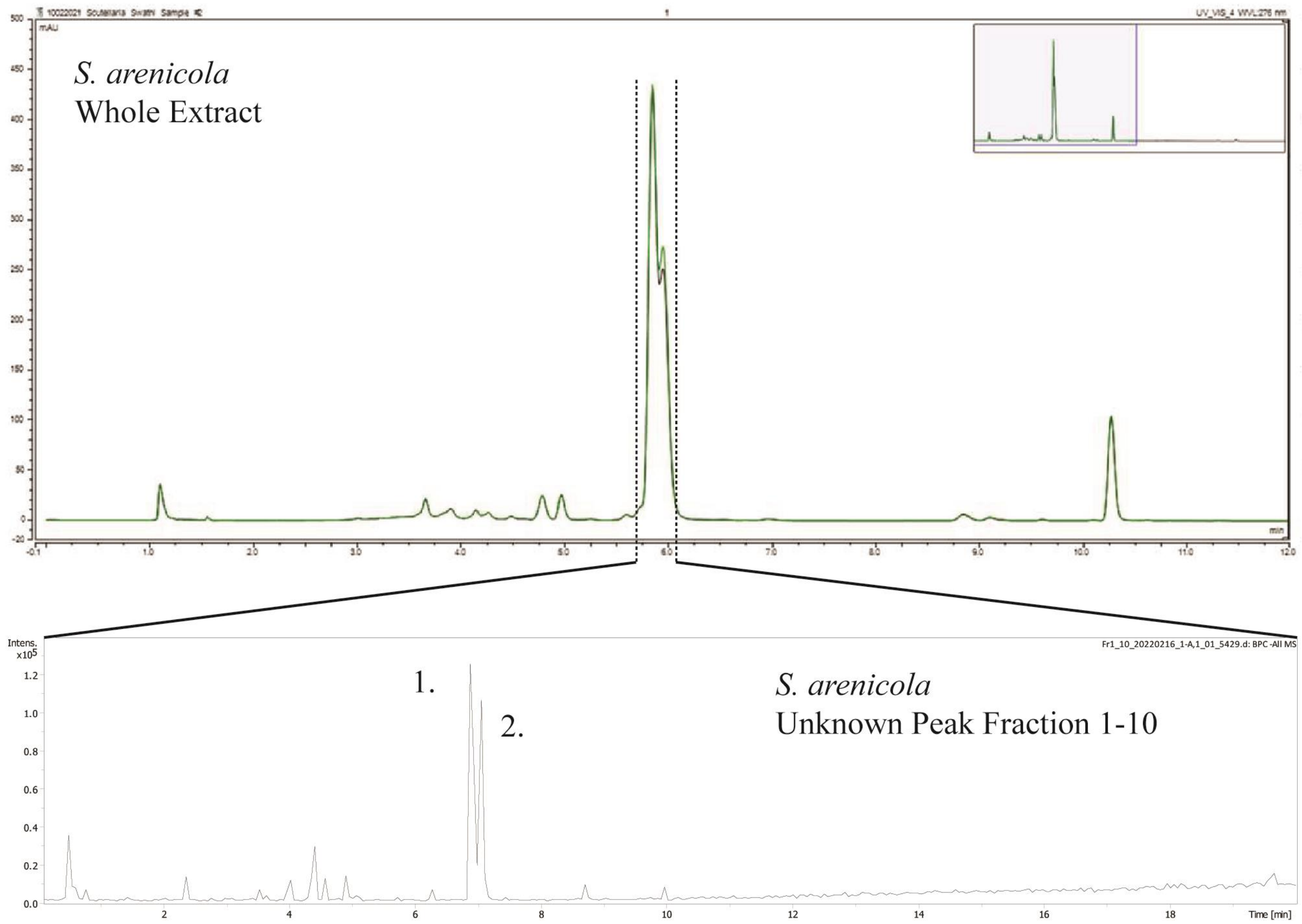

B)

1. Luteolin-7-O-glucuronide

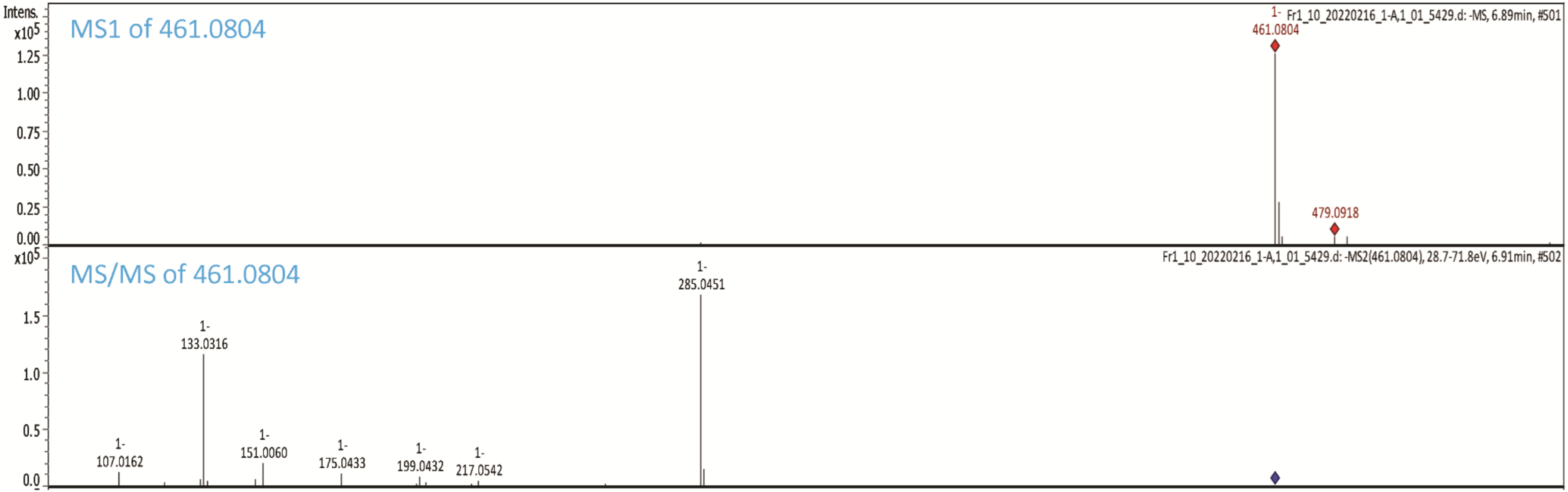

C)

2. 5,7,2’-trihydroxy-6-methoxyflavone 7-O-glucuronide

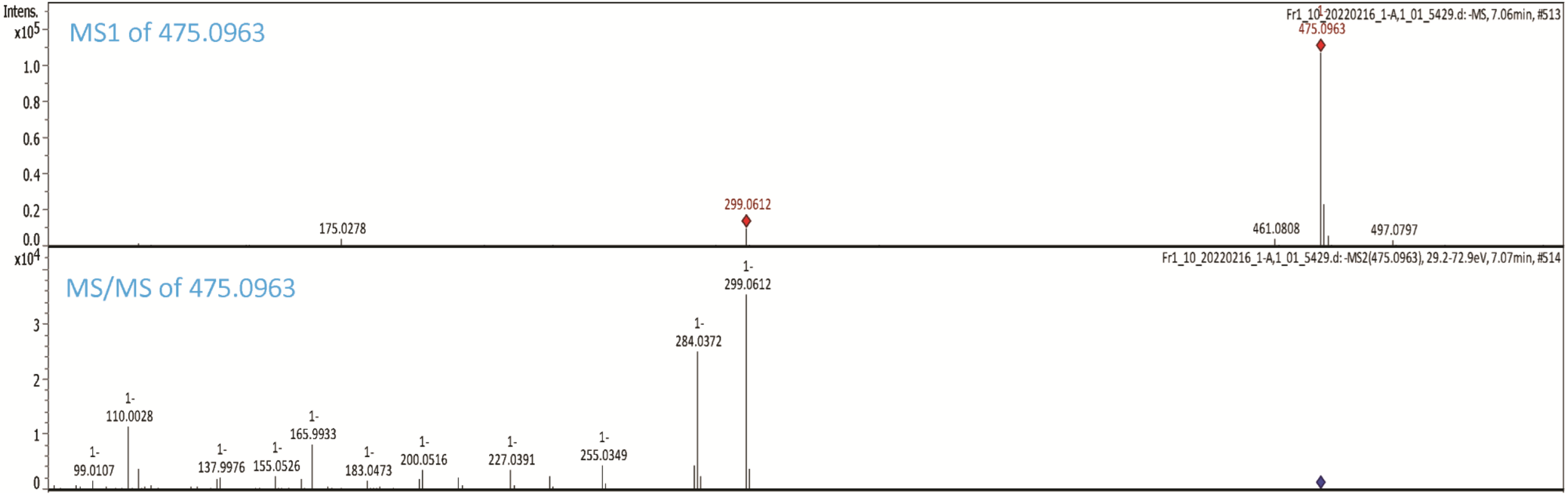

Fig S5. HPLC, Fractionation, and LC-MS/MS analysis for identification of *S. baicalensis* unknown compounds fraction 2-11. A) HPLC chromatogram of the whole extract and chromatogram of fraction collected from unknown major peaks in whole extract as indicated. 1, and 2 represent the metabolites identified whose MS/MS data is presented. B) MS1 spectrum of hydrogenated scutellarin showing accurate precursor mass and MS2 spectrum of the precursor. C) MS1 spectrum of a scutellarin isomer showing accurate precursor mass and MS2 spectrum of the precursor.

A)

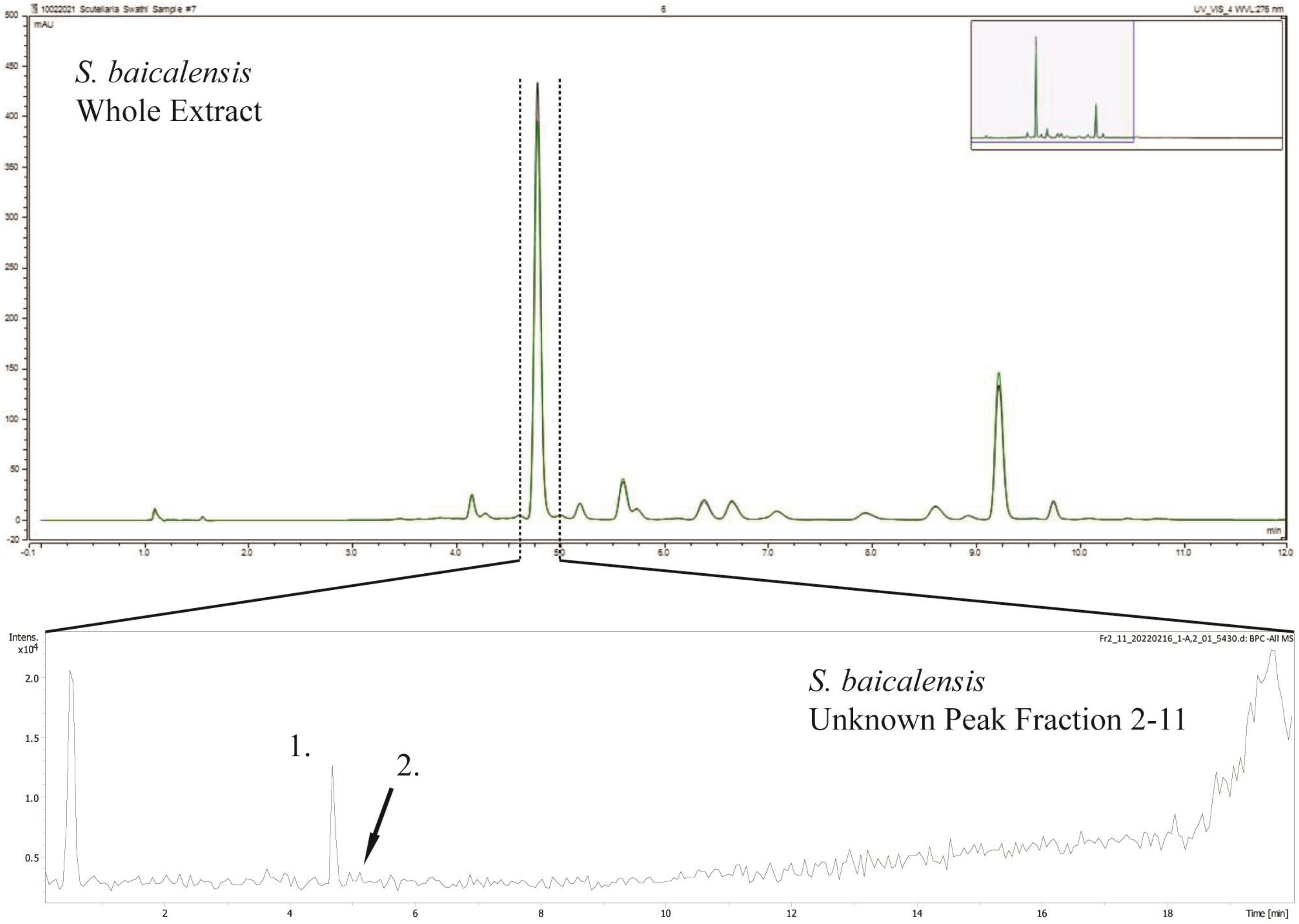

B) 1. Hydrogenated Scutellarin

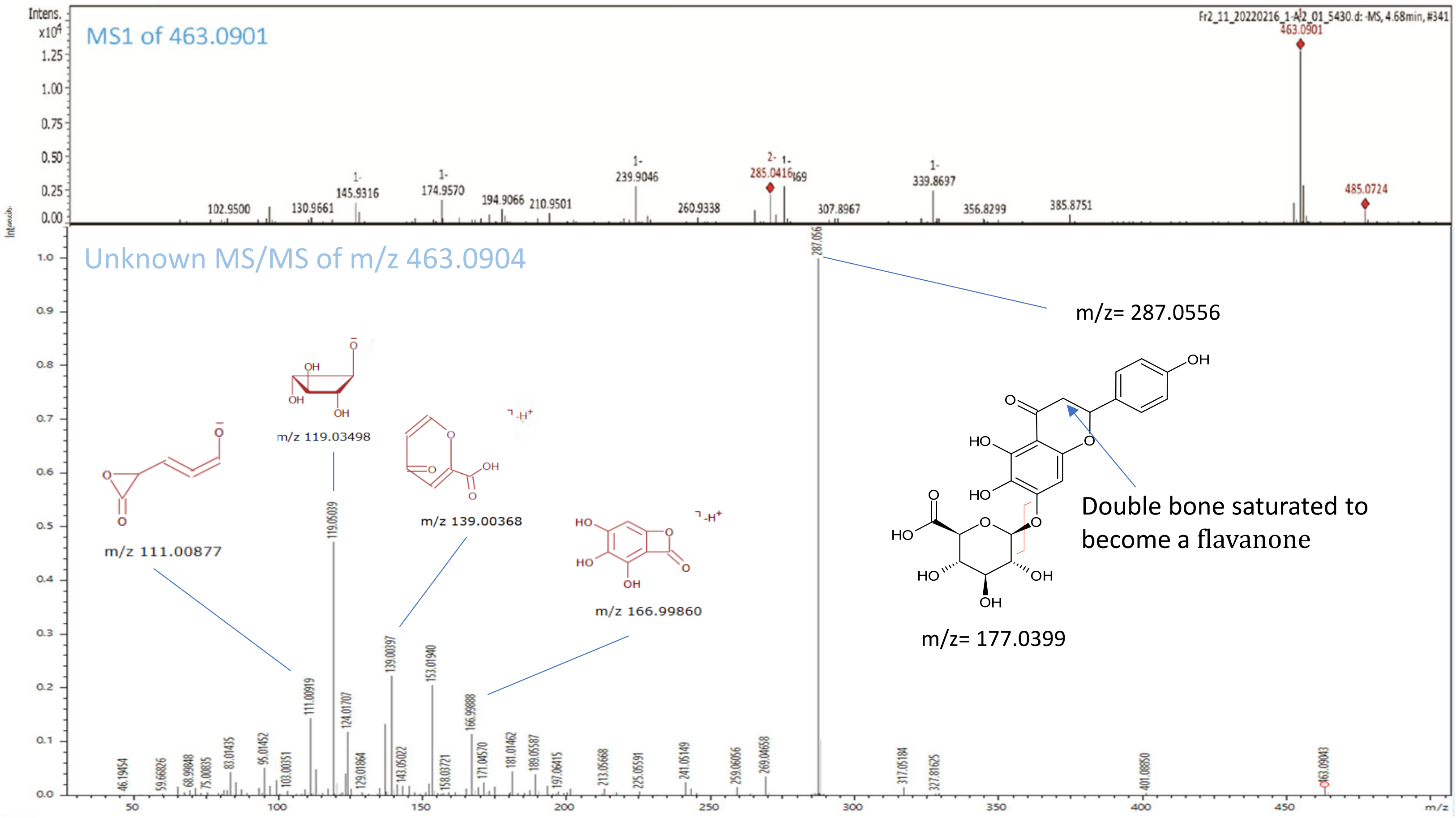

C) 2. Scutellarin Isomer

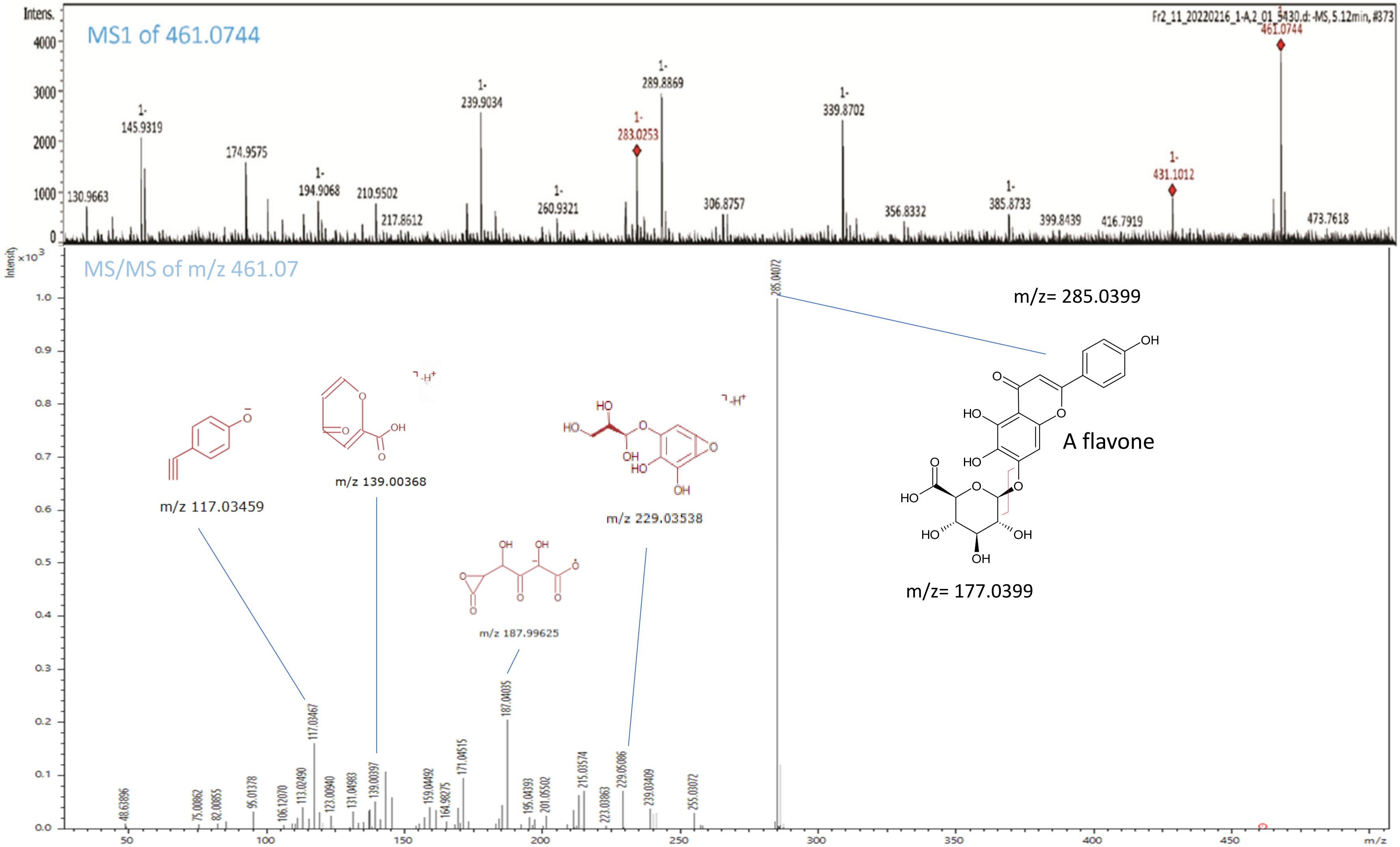

Fig S6. HPLC, Fractionation, and LC-MS/MS workflow for identification of *S. baicalensis* unknown compounds fraction 2-12. A) HPLC chromatogram of the whole extract and chromatogram of fraction collected as indicated. 1, 2 and 3 represent the metabolites identified whose MS/MS data is presented. B) MS1 spectrum of hydrogenated scutellarin showing accurate precursor mass and MS2 spectrum of the precursor in both dimeric and monomeric forms. C) MS1 spectrum of scutellarin isomer showing accurate precursor mass and MS2 spectrum of the precursor. D) MS1 spectrum of apigenin-7-O-glucuronide showing accurate precursor mass and MS2 spectrum of the precursor.

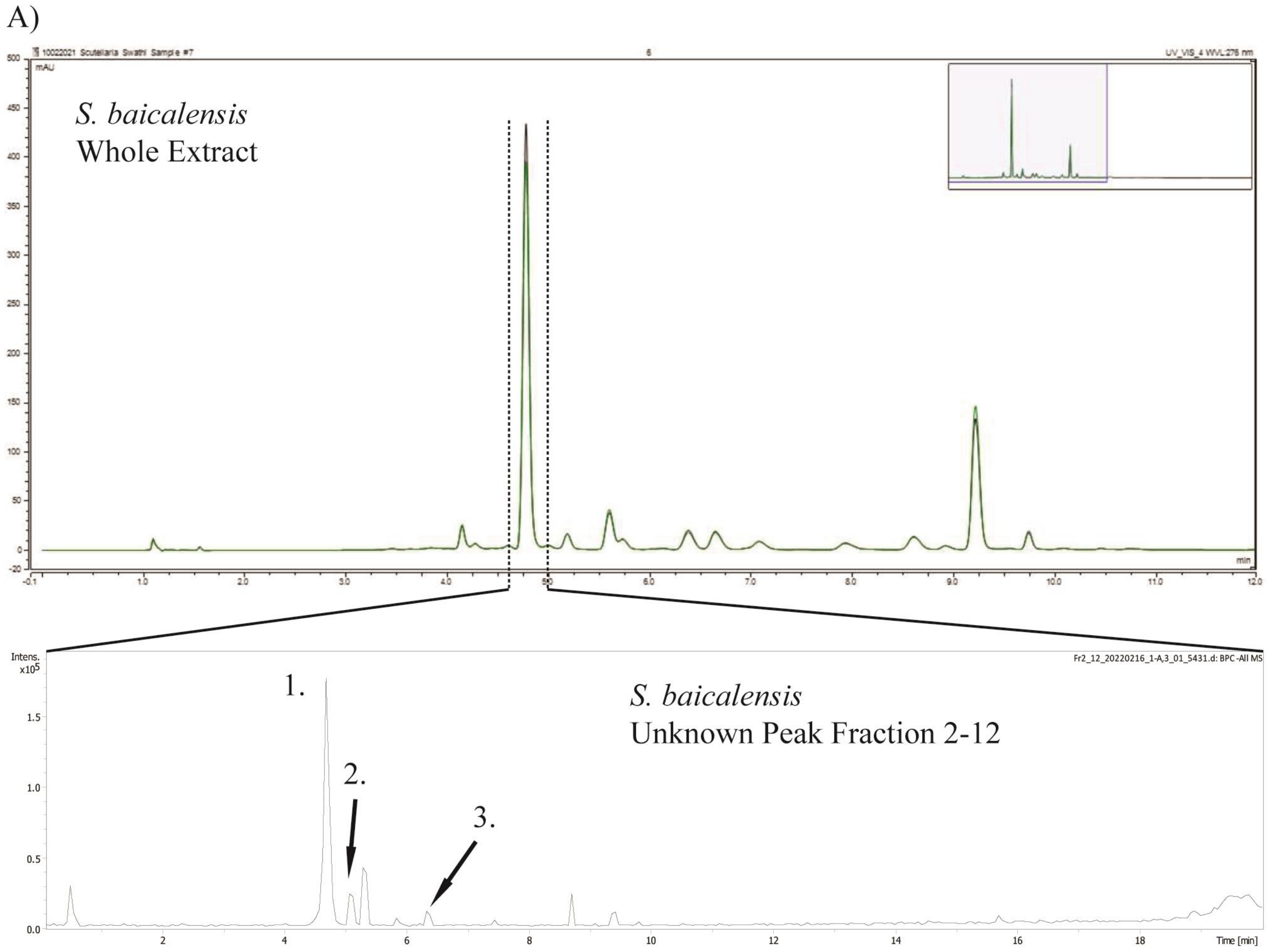

B)

1. Hydrogenated Scutellarin (dimeric form)

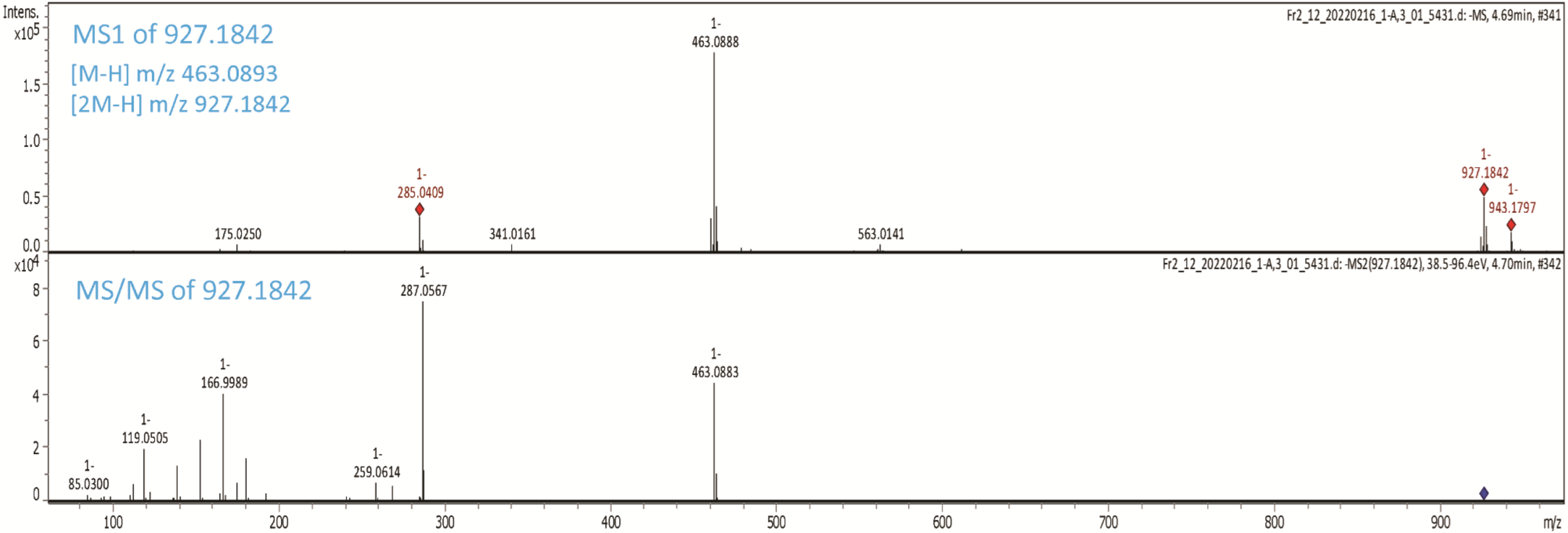

1. Hydrogenated Scutellarin (monomeric form)

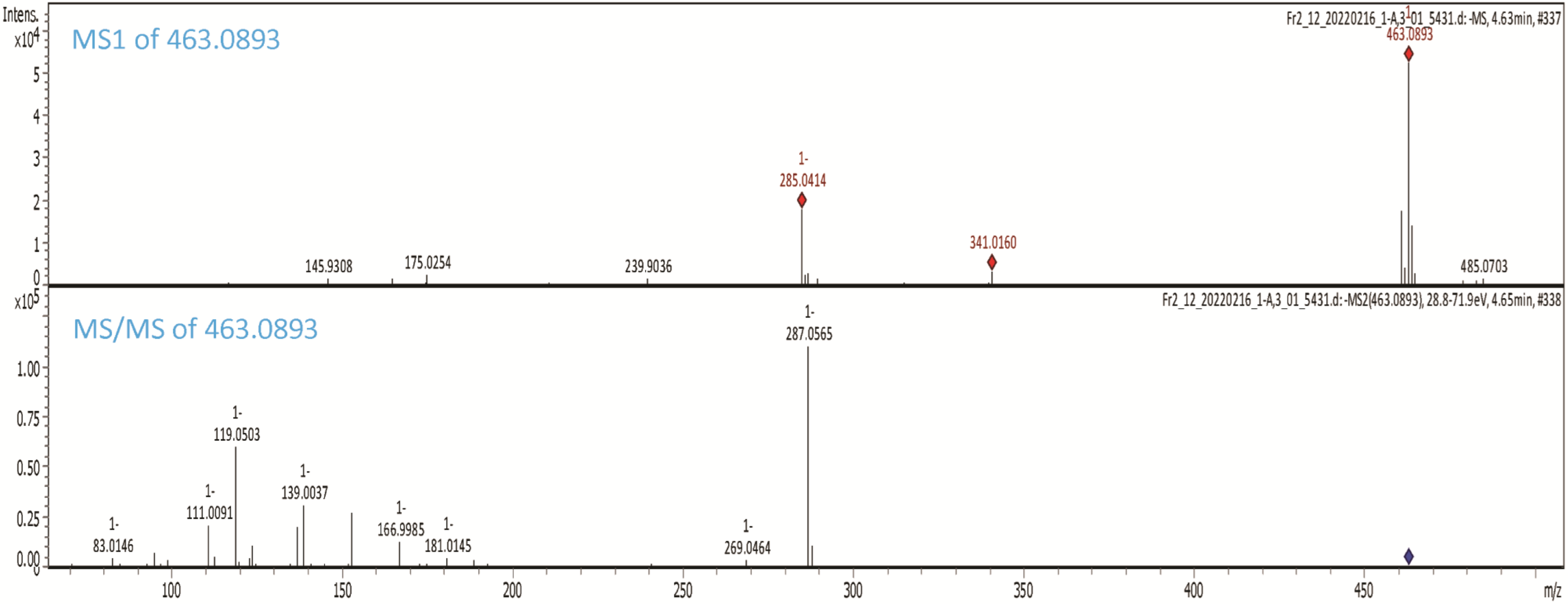

C)

2. Scutellarin isomer

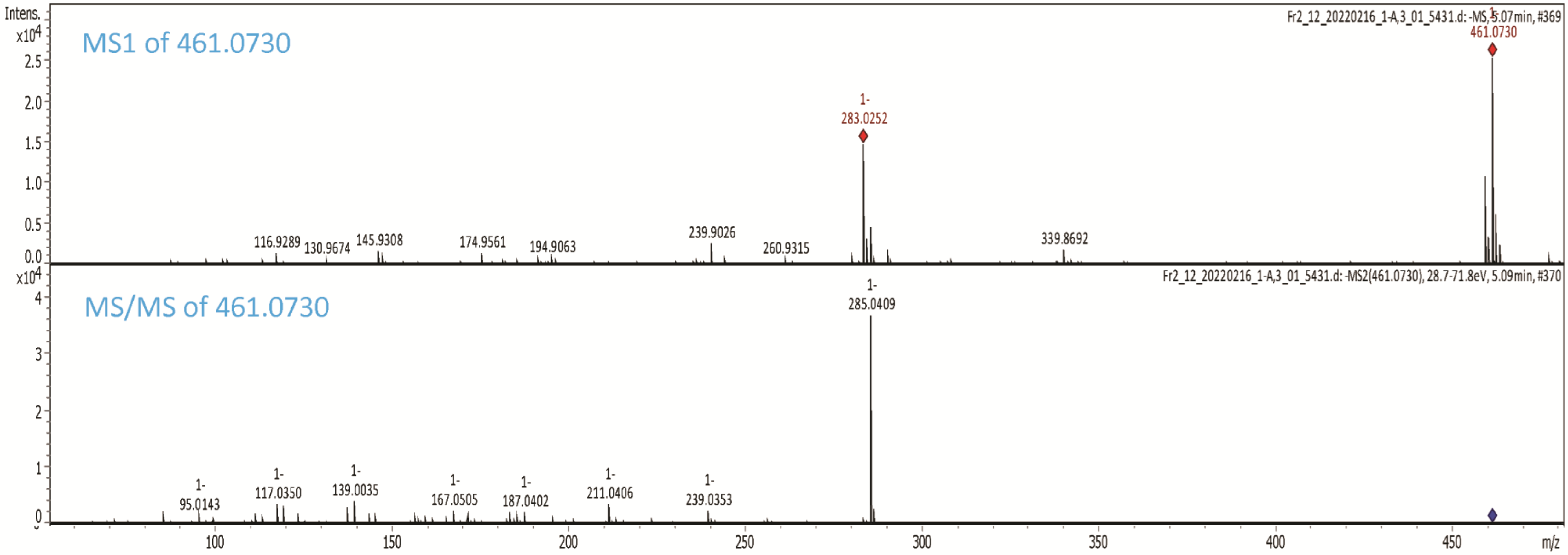

D)

3. Apigenin-7-O-glucuronide

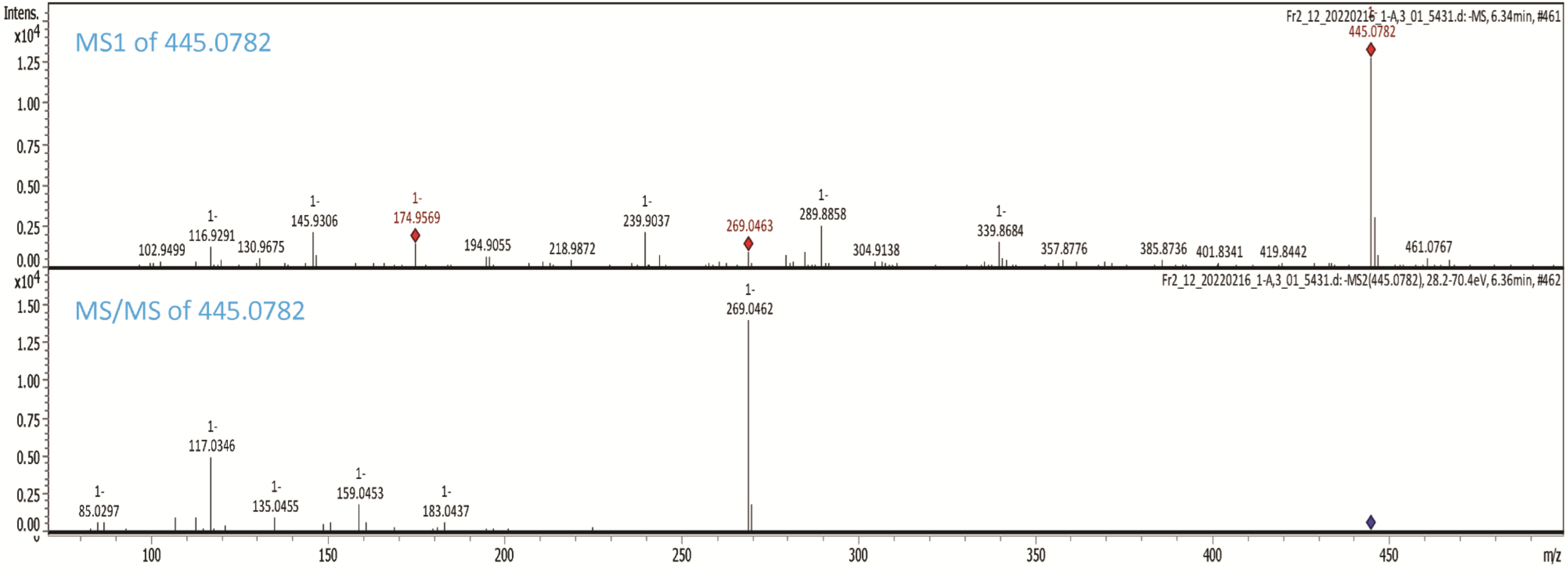

Supplement: Supplementary file 1 — Supplementary Information. [file 41598_2022_17586_MOESM1_ESM.pdf]
